# Generation and Reactivity of a High-Spin Iron(IV)-Oxo Complex That Is Stable at Ambient Temperatures

**DOI:** 10.1021/jacs.5c00503

**Published:** 2025-04-16

**Authors:** Christopher D. Hastings, Lucy S. X. Huffman, William W. Brennessel, Brandon R. Barnett

**Affiliations:** Department of Chemistry, University of Rochester, Rochester, New York 14627, United States

## Abstract

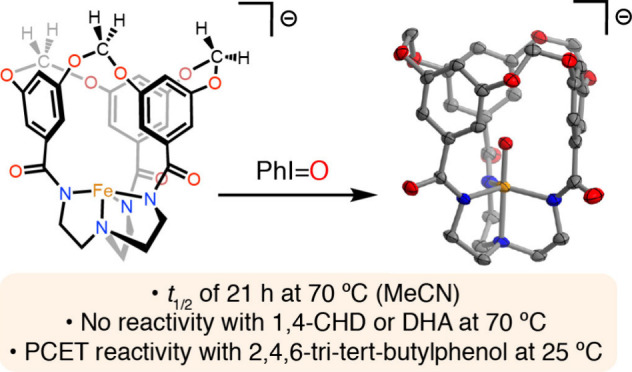

Nature operates a variety of challenging oxidation reactions through intermediates bearing tetravalent iron centers bound to a terminal oxo ligand. The high-spin (*S* = 2) electronic configuration is believed to be particularly important in C–H activation reactions mediated by iron(IV)-oxo species. Coordination environments that promote high-spin ground states obviate the need for spin-state crossing to access this state and can promote rapid oxidation reactivity. As a result, however, synthetic iron(IV)-oxo species with *S* = 2 ground states tend to exhibit poor thermal stabilities, which has hampered a broader elucidation of their reactivity profiles. In this work, we report the synthesis of a remarkably stable high-spin iron(IV)-oxo complex that localizes the Fe=O unit within a rigid organic macrocycle. This design results in essentially unlimited stability at ambient temperatures and a half-life of 21 h at 70 °C in CH_3_CN, endowing this compound with the highest thermal stability for a high-spin Fe^IV^=O complex reported to date. The ligand’s steric profile shuts down intermolecular reactivity with potential O atom acceptors and hydrocarbons bearing weak C–H bonds, but proton-coupled electron transfer reactivity with 2,4,6-tri-*tert*-butylphenol (TTBP) occurs readily at room temperature despite its steric bulk.

The impressive activities and selectivities exhibited by many native metalloenzymes are connected to their abilities to confine reactive species within well-defined microenvironments.^1^ Obviating deleterious reactions of high-energy intermediates is accomplished, in part, by isolating the active site from the exterior environment. Hydrophobic tunnels and channels seen in certain metalloenzymes can facilitate substrate/product transport to/from the active site, and also may assist in properly orienting substrates for reactivity.^2,3^

Transient terminal oxo (O^2–^) complexes of iron mediate a variety of biologically important oxidation reactions involving formal *O*-atom transfer to a substrate (e.g., C–H hydroxylation; water oxidation).^4−7^ Computational work has suggested that the high-spin electronic state (*S* = 2) of iron(IV)-oxo compounds can have lower intrinsic activation barriers compared to the intermediate-spin (*S* = 1) state, although experimentally probing this notion remains challenging.^8^ Weak ligand fields and/or trigonal coordination environments promote high-spin ground states for iron(IV)-oxo species, with a small handful of such compounds having been characterized.^9−17^ Owing to their proclivities to homolytically cleave C–H bonds from either the ligand backbone or solvent, manipulation is usually confined to low temperatures, which has complicated a broader establishment of their reaction chemistries and solution-phase properties.

In an effort to kinetically stabilize reactive metal-bound functional groups within a soluble molecular platform, our group has introduced a chelating ligand that localizes a rigid void around an open coordination site.^18^ Starting from tris(2-aminoethyl)amine (TREN), a macrocyclization reaction links together three aryl rings in a manner reminiscent of Cram’s cavitands.^19^ In our initial report, we demonstrated that the rigidity and narrow confines of this macrocycle yield a one-dimensional channel that controls access to the metal coordination sphere.^18^ Herein, we show that our ligand scaffold enables the generation of a high-spin iron(IV)-oxo compound via *O*-atom transfer to the trigonal monopyramidal ferrous species [FeL^OCH2O^]^−^ ([**1**]^−^). The five-coordinate ferryl oxo [Fe(O)L^OCH2O^]^−^ ([**2**]^−^) has been characterized using single-crystal X-ray diffraction as well as through a variety of spectroscopic techniques. Owing to the rigid macrocycle guarding the Fe=O unit, [**2**]^−^ displays virtually unlimited kinetic persistence in solution at ambient temperatures, only undergoing thermal decay at elevated temperatures.

Treatment of an *N,N*-dimethylacetamide (DMA) solution of H_3_L^OCH2O^ with *in situ*-generated potassium tris(hexamethyldisilazido)ferrate(II)^20^ yields K[FeL^OCH2O^] (K[**1**]) as a colorless solid (Figure 1a). To improve solubility and facilitate purification, this crude salt was treated with [2.2.2]cryptand to generate [K(Crypt)][**1**], which could be isolated via crystallization in 43% yield. The solid-state structure as determined from X-ray diffraction reveals a trigonal monopyramidal geometry (τ_4_ = 0.84)^21^ with the open axial coordination site localized within the macrocycle-enforced void (Figure S29). Magnetic moment determination via Evans method (5.6 μ_B_) is consistent with a *d*^6^ high-spin (*S* = 2) electronic configuration on account of the small *d*-orbital splittings produced by the tris(amidyl)amine ligand.^22−24^

**Figure 1:**
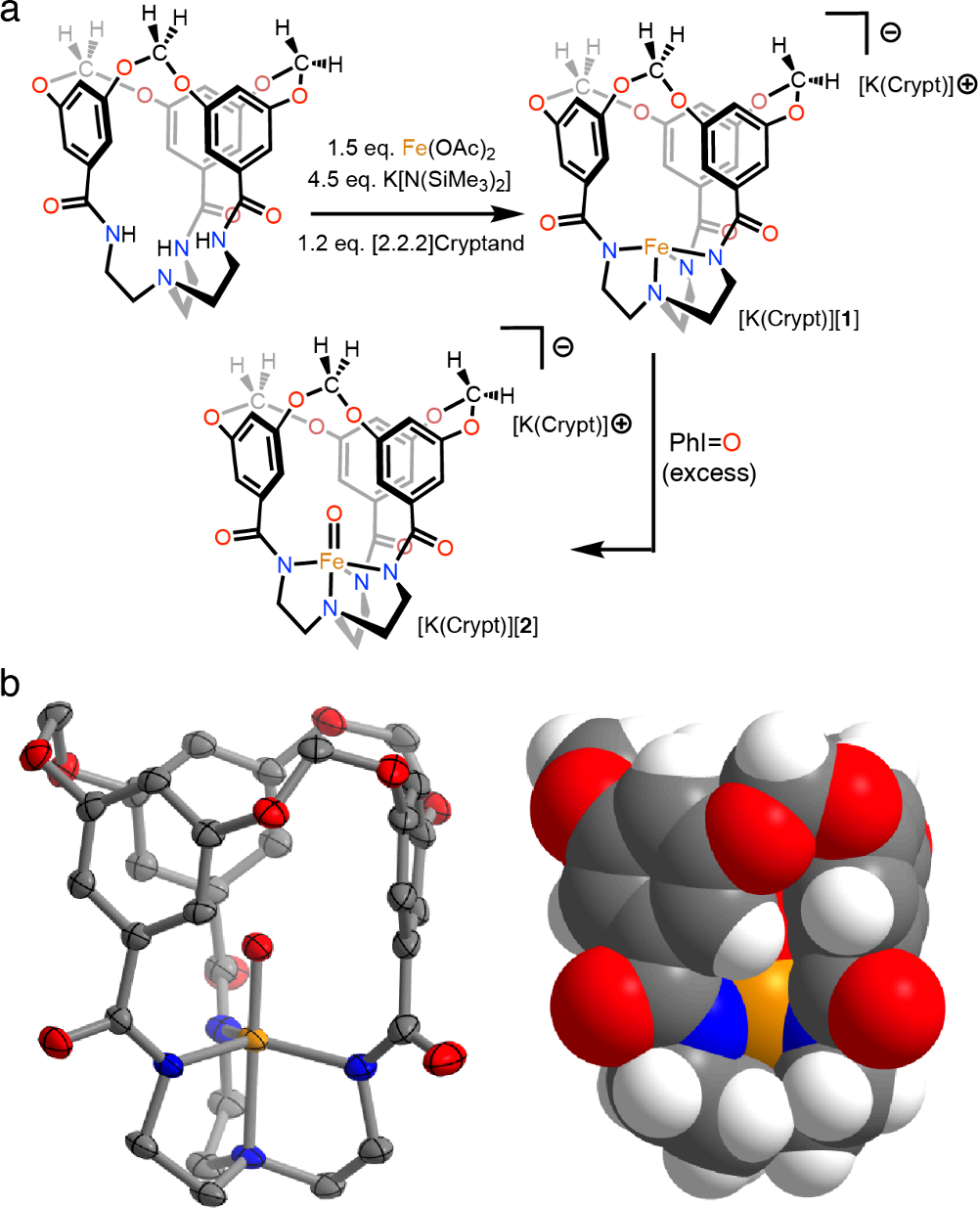
a) Syntheses of [K(Crypt)][**1**] and [K(Crypt)][**2**]. b) Solid-state structure of the anionic component of [K(Crypt)][**2**] showing both thermal ellipsoids (50% probability level) and a spacefilling model.

Treatment of [K(Crypt)][**1**] with iodosylbenzene (PhIO) in acetonitrile begins to generate an orange/yellow solution within seconds. Electronic absorption spectra of the resulting compound (Figure S4) display a new peak at 378 nm (ε = 5300 M^–1^ cm^–1^) with a shoulder at approximately 450 nm (ε = 760 M^–1^ cm^–1^), as well as a broad and weak feature with a λ_max_ at 875 nm (ε = 61 M^–1^ cm^–1^). This latter feature aligns well with the *d*–*d* bands seen in known high-spin ferryl oxo complexes.^9,10^ Single crystals suitable for X-ray diffraction were grown by allowing diethyl ether to diffuse into a DMA solution and confirm this product to be the trigonal bipyramidal terminal oxo compound [K([2.2.2]cryptand)][Fe(O)L^OCH2O^] ([K(Crypt)][**2**]; Figure 1a-b). The presence of an intracavity oxo ligand is supported by the short Fe=O bond length of 1.6415(19) Å. The oxo *O*-atom occupancy freely refines to 1.0, thereby ruling out cocrystallization of the product with remnant [**1**]^−^. Infrared spectra of [**2**]^−^ display a stretch at 859 cm^–1^ that moves to 823 cm^–1^ upon ^18^O labeling of the oxo ligand, with both values being comparable to those seen in other ferryl oxo species (Figure S5).^6^ Evans method measurements return an effective magnetic moment (5.4 μ_B_) in accord with a high-spin (*S* = 2) configuration, as expected owing to the trigonally symmetric coordination environment.

Ferryl oxo complexes that adopt high-spin ground states usually display poor thermal stabilities in solution, with intramolecular C–H activation of the supporting ligand scaffold often being operative at or below ambient temperatures.^24,25^ Interestingly, acetonitrile solutions of [**2**]^−^ show no change in their electronic spectra upon standing at room temperature for weeks, and only begin to change upon heating (Figure 2a). Consumption of [**2**]^−^ is most easily followed by monitoring the disappearance of the 450 nm shoulder in the electronic spectrum (Figures S11–12). At 70 °C, [**2**]^−^ displays a half-life of 21 h and is, to our knowledge, the most thermally stable high-spin ferryl oxo reported to date.^26^ We attribute the kinetically persistent nature of [**2**]^−^ in solution to the rigidity of the macrocyclic [L^OCH2O^]^3–^ ligand, which credibly inhibits both intra- and intermolecular reactivity. Structurally, an interesting point of comparison is Chang’s trigonal bipyramidal [Fe(O)tpa^Ph^]^−^, which decays readily at −40 °C via *ortho* C–H activation of a flanking phenyl ring to form a ferric phenoxide.^13,24^ Analogous reactivity with the *ortho* C–H bonds in [**2**]^−^ could be envisaged but would require rotation of the corresponding ring to facilitate formation of a new O–C σ-bond. Accordingly, the persistent and rigid steric profile of the macrocycle-defined cavity in [L^OCH2O^]^3–^ is very likely key to the stability displayed by [**2**]^−^.

**Figure 2:**
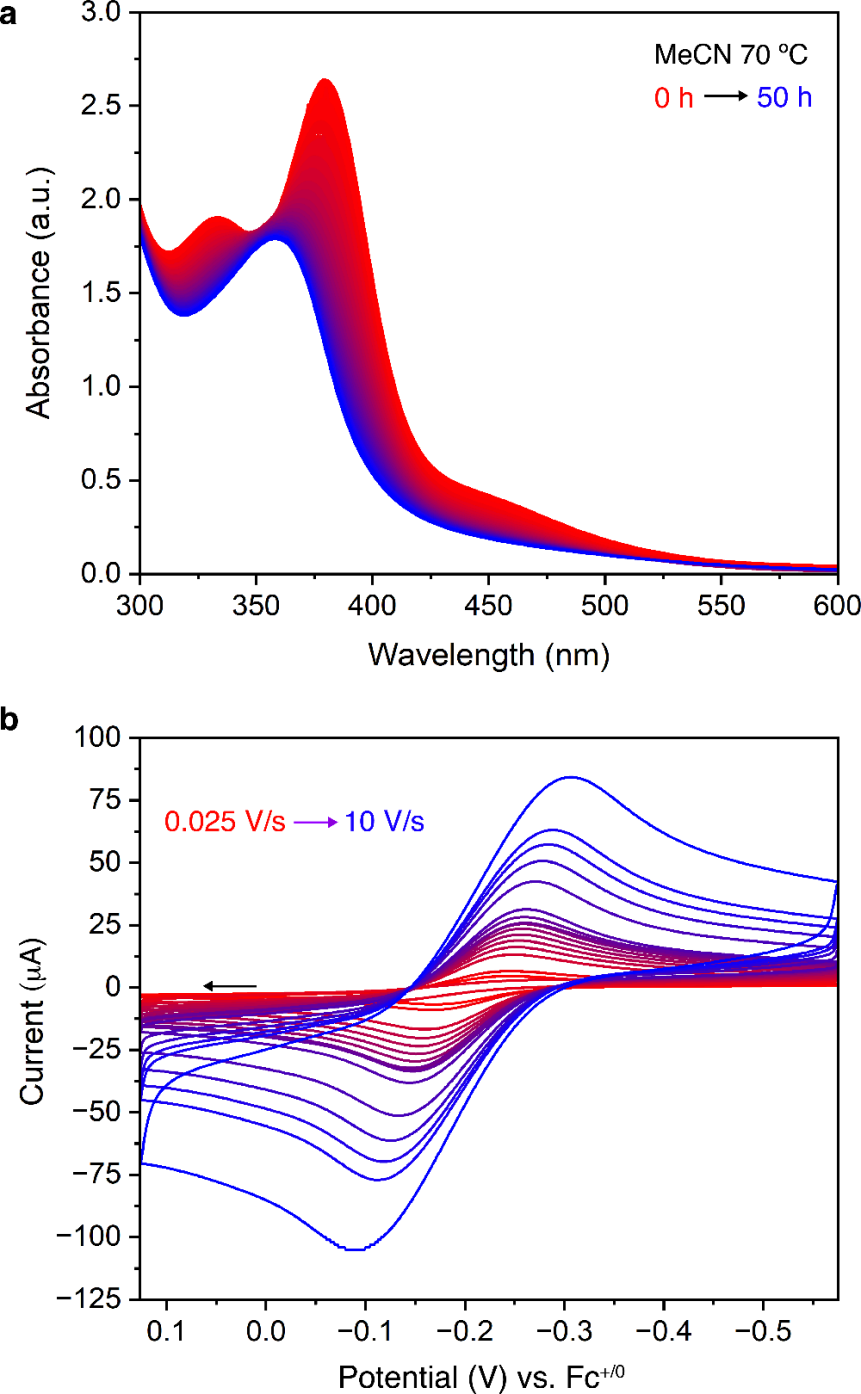
a) Electronic spectra of [**2**]^−^ in acetonitrile at 70 °C monitored over 50 h, with spectra acquired at 5 min intervals. The time dimension is depicted with a red-to-blue gradient. b) Cyclic voltammetry measurements of [**2**]^−^ in DMA at different scan rates showing the electrochemically quasi-reversible feature assigned as the Fe^IV/III^ couple. The arrow denotes the scan direction.

The kinetic persistence of [**2**]^−^ facilitates solution-phase characterization using techniques often inaccessible to high-spin ferryl oxo species. For example, ^1^H NMR has proven to be a reliable method to identify [**2**]^−^, which gives rise to broadened and paramagnetically shifted but easily identifiable peaks (Figure S2). Additionally, cyclic voltammetry allows for the identification of the redox potential corresponding to the Fe^IV^/Fe^III^ couple, which appears as an electrochemically quasi-reversible feature at −0.20 V vs Fc^+/0^ in DMA (Figure 2b).^27^ The Fe^IV^/Fe^III^ couple for [**2**]^−^ appears at a similar potential as does Que’s [Fe(O)TMG_3_tren]^2+^ (indirectly determined as +0.27 V vs SCE or approximately −0.2 V vs Fc^+/0^ in CH_2_Cl_2_)^28,29^ but is anodically shifted from that in Borovik’s [Fe(O)H_3_buea]^−^ (−0.90 V vs Fc^+/0^).^30^ The large difference in Fe^IV/III^ potentials between anionic [**2**]^−^ and [Fe(O)H_3_buea]^−^ can be ascribed to the effects of H-bonding interactions between the urea N–H bonds and the oxo ligand in the latter complex. These secondary sphere interactions lead to a decrease in covalency in the iron-oxo bonding interaction as determined previously,^15^ and further supported by the longer Fe=O bond distance in [Fe(O)H_3_buea]^−^ (1.6804(7) Å).^10^ Additionally, Density Functional Theory calculations (B3LYP, def2-TZVPP) yield results consistent with a higher degree of Fe=O covalency in [**2**]^−^, including a larger spin density (ρ_O_) and smaller charge magnitude on the oxo *O*-atom in comparison to [Fe(O)H_3_buea]^−^ (Table 1). In contrast, single-point calculations on dicationic [Fe(O)TMG_3_tren]^2+^ yield almost identical ρ_O_ and charge values for the oxo *O*-atom as those in [**2**]^−^. While it should be noted that DFT can struggle to provide ρ_O_ quantities in agreement with experimentally determined values,^31^ the qualitative trends observed here are likely valid given that all computations were carried out using identical parameters. Additionally, we note that our results mirror previous computational findings for [Fe(O)TMG_3_tren]^2+^ and [Fe(O)H_3_buea]^−^.^31,32^

**Table 1:** Comparative Iron-Oxo Bond Distances (from XRD) and DFT-Calculated Charges and Spin Populations (ρ) for the Oxo O-Atoms

Complex	*d*(Fe=O) (Å)	O_oxo_ Charge	ρ_O_
[**2**]^−^	1.6415(19)	–0.51	0.64
[Fe(O)H_3_buea]^−^	1.6804(7)^10^	–0.66	0.42^a^
[Fe(O)(TMG_3_tren)]^2+^	1.661(2)^25^	–0.50	0.63

a The experimentally determined value from EPR measurements is 0.56.^31^

Presumably owing to the steric profile of the [L^OCH2O^]^3–^ ligand, [**2**]^−^ shows no sign of *O*-atom transfer reactivity upon exposure to excess trimethylphosphine (PMe_3_), styrene, or ethylene (C_2_H_4_; Scheme 1 and Figures S13–16). Additionally, intermolecular C–H cleavage of 1,4-cyclohexadiene (CHD) is sufficiently sluggish such that consumption of [**2**]^−^ at 70 °C in MeCN is not appreciably altered by its addition (10 equiv; Figures S17–18). Monitoring the reaction of [**2**]^−^ with an excess of either CHD or 9,10-dihydroanthracene (DHA) at 70 °C results in negligible consumption of the hydrocarbon reactant and no observation of the expected products of PCET reactivity (benzene or anthracene, respectively; Figures S19–22). Comparatively, Que’s highly encumbered [Fe(O)(TMG_3_tren)]^2+^ reacts with these reagents at −30 °C to yield benzene and anthracene, respectively.^9^ We note that DFT calculations support the notion that the PCET reactions of [**2**]^−^ with these reagents should be highly exergonic. At the B3LYP/def2-TZVPP level, the O–H bond in the incipient ferric hydroxide resulting from HAT is computed to have a bond dissociation free energy (BDFE) of 91 kcal/mol,^33^ which is substantially larger than the C–H BDFEs in these hydrocarbon substrates.^34^ Accordingly, the lack of substantial reactivity with CHD and DHA can be ascribed to steric hindrance. Judging from the structure of [**2**]^−^, attack of a C–H bond at the Fe=O π* orbital would appear to be blocked by the encapsulating aryl rings.^35^ Attack via the Fe=O σ* orbital would require substrate ingress through the narrow macrocycle aperture, which has previously been shown to inhibit entry of the much smaller acetonitrile molecule.^18^

**Scheme 1:**
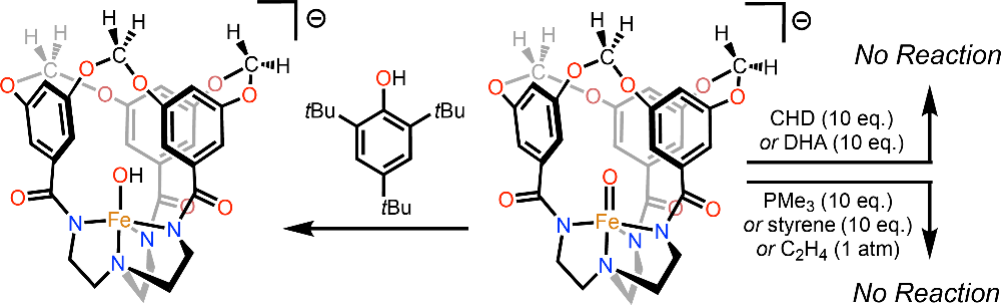
Reactions of [2]^−^ with Potential *O*-Atom Acceptors or *H*-Atom Donors All reactions were carried out in acetonitrile solvent.

Despite the recalcitrance of [**2**]^−^ to react via PCET with CHD or DHA, reactivity with 2,4,6-tri-*tert*-butylphenol (TTBP; O–H BDFE in MeCN = 74.8 kcal/mol)^34^ proceeds readily at 25 °C (Scheme 1 and Figure S24). Electron paramagnetic resonance (EPR) spectra of this solution suggest the formation of a non-integer spin complex showing broad features at nearly identical *g*_eff_ values as the *S* = 5/2 trigonal bipyramidal ferric hydroxide [Fe(OH)H_3_buea]^−^ (Figure 3).^36^ We assign this product as the ferric hydroxide [Fe(OH)L^OCH2O^]^−^, which can also be generated via hydroxide addition to the previously reported iron(III) compound FeL^OCH2O^ (see the Supporting Information).^37^ Importantly, the EPR spectrum of independently synthesized [Fe(OH)L^OCH2O^]^−^ matches the broad features seen in the spectra following the reaction of [**2**]^−^ with TTBP (Figure 3).

**Figure 3:**
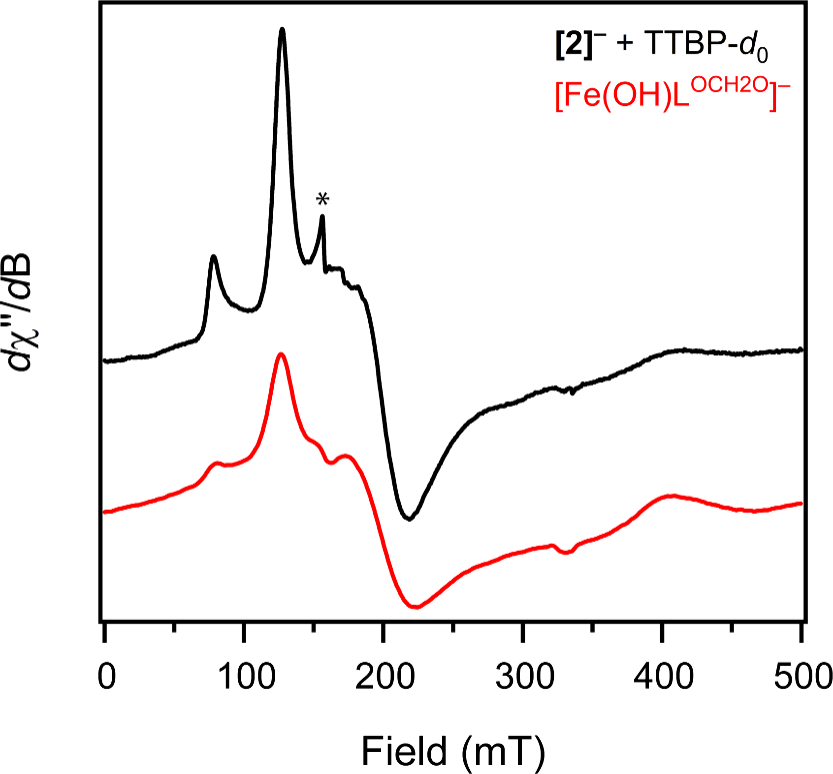
X-band EPR spectra (10 K) of solutions comprising the reaction of [**2**]^−^ with TTBP (black) and the independently generated iron(III) hydroxide [Fe(OH)L^OCH2O^]^−^. The peak labeled with an asterisk (*) corresponds to a small contaminant at *g* ≈ 4.3.

Owing to the steric profiles of [**2**]^−^ and TTBP, and to the absence of a reaction between [**2**]^−^ and CHD or DHA, we posit that a concerted PCET mechanism is unlikely. Spectroscopic monitoring of reaction kinetics paints a picture that is consistent with a stepwise proton transfer-electron transfer (PT-ET) reaction mechanism. Pseudo-first-order rate constants were abstracted at early time points from reactions run with either *O*-deuterated (TTBP-*d*_1_) or nondeuterated (TTBP-*d*_0_) substrate, yielding a primary kinetic isotope effect (KIE) of 3.0 (Figures S24–27). This value is consistent with rate-limiting proton transfer to give an incipient protonated version of the ferryl oxo and a phenoxide anion.^38^ Reactions with either TTBP-*d*_0_ or TTBP-*d*_1_ slow significantly after the earliest time points, yielding a profile that we believe to be reflective of an equilibrium isotope effect in the initial proton transfer step, although analysis is complicated by the reaction of the incipient TTBP radical with [**2**]^−^ (Figure S28). Future experiments will probe this reaction sequence in greater detail and will interrogate the use of [**2**]^−^ and its unique steric profile for synthetically useful transformations.

## Supporting Information Available

Synthetic procedures and characterization data (NMR, electronic, and vibrational spectroscopies; cyclic voltammetry) for all newly reported compounds; details of kinetics measurements and additional supporting data; details of density functional theory calculations; details of crystallographic structure determinations (PDF)
