# Supplementary material for: Generation and Reactivity of a High-Spin Iron(IV)-Oxo Complex That Is Stable at Ambient Temperatures
Source: J Am Chem Soc. 2025 Apr 16;147(17):14031–5. doi: 10.1021/jacs.5c00503 (PMC12046554; doi:10.1021/jacs.5c00503)
Supplement: Supplementary file 1 — ja5c00503_si_001.pdf [file ja5c00503_si_001.pdf]

Supporting Information for the Paper Entitled:

Generation and Reactivity of a High-Spin Iron(IV)-Oxo Complex that is Stable at Ambient Temperatures

Christopher D. Hastings, Lucy S. X. Huffman, William W. Brennessel, and Brandon R. Barnett*

Department of Chemistry, University of Rochester, Rochester, NY 14627

Email: brandon.barnett@rochester.edu

Contents

S1. General Considerations – p. 2

S2. Syntheses and Characterization – p. 3

S3. Kinetic Measurements of Self-Decay and Attempted Reactions with CHD, DHA, and Potential O-atom Acceptors – p. 10

S4. Reaction of [**2**]$^{-}$ with TTBP – p. 21

S5. Details of Density Functional Theory Calculations – p. 24

S6. Details of Crystallographic Structure Determinations – p. 26

S7. References – p. 31

S1. General Considerations

All manipulations were carried out under an atmosphere of purified dinitrogen using standard Schlenk and glovebox techniques. Unless otherwise stated, reagent-grade starting materials were purchased from commercial sources and either used as received or purified by standard procedures.[1] The proligand H_3L^{OCH2O} was synthesized as reported previously.[2] Iodosylbenzene-^{18}O ($PhI^{18}O$) was prepared through an exchange reaction between non-labelled PhIO and ^{18}O-labelled water as reported elsewhere.[3] The tri(2,4,6-*tert*-butyl)phenoxy radical and unlabeled iodosylbenzenewere synthesized as previously reported.[4,5] Unless otherwise stated, organic solvents were deoxygenated and dried using a Pure Process Technologies solvent purification system. *N,N*-dimethylacetamide (DMA) was stored over activated 3 Å molecular sieves, transferred via cannula into a separate flask, sparged with N_2 for 1 h, and stored over fresh 3 Å molecular sieves in the glovebox prior to use. Acetonitrile-d_3 was dried by distillation over CaH_2 under N_2, degassed via three freeze-pump-thaw cycles, and stored in the glove box over activated neutral alumina. Molecular sieves (3 Å), neutral alumina and Celite were separately pre-activated in a 180 ºC oven overnight, then transferred into a round bottom flask and heated under vacuum ($P < 100$ mTorr) at a temperature in excess of 200 ºC for at least 12 h, and then stored in the glovebox. Tetra(*n*-butylammonium) hexafluorophosphate (electrolyte for electrochemical measurements) was recrystallized three times from ethanol, and then dried under vacuum with P_2O_5 at 120 ºC until the pressure reached 50 mTorr.

Solution ^1H and $^{31}P\{^1H\}$ nuclear magnetic resonance (NMR) spectra were recorded on a JEOL 500 MHz spectrometer locked on the signal of deuterated solvents. ^1H chemical shifts are reported in ppm relative to $SiMe_4$ ($\delta = 0.0$ ppm) with reference to residual solvent resonances. ^{31}P chemical shifts are reported in ppm relative to an 85% H_3PO_4 solution ($\delta = 0.0$ ppm) and were referenced externally. Attenuated total reflectance infrared (ATR-IR) spectra were recorded on a PerkinElmer Spectrum 3 infrared spectrometer within a glovebox. Electronic absorption measurements were recorded using an Agilent Cary 6000i UV-Vis-NIR spectrometer. Samples were prepared in the glovebox and sealed in a quartz cuvette (1 cm pathlength). X-band electron paramagnetic resonance (EPR) measurements were carried out on a Bruker EMXplus spectrometer (microwave frequency of 9.382 GHz). Samples were prepared as solutions in MeCN in the glovebox, glassed via flash-cooling in liquid nitrogen, and loaded into the spectrometer. Electrospray ionization mass spectrometry (ESI-MS) measurements were performed on a Thermo Scientific LTQ Velos ion trap instrument. High-resolution mass spectrometry (ESI) measurements were performed on a Dionex Ultimate 3000 connected to a Q Exactive Plus mass spectrometer (Thermo Fisher) without the use of a column. Electrochemical measurements were performed using a CH Instruments 620 D potentiostat with a three electrode setup, including a $Ag/AgNO_3$ (1 M) reference electrode (CHI111), a Pt wire counter electrode (CHI115, surface area in solution of 0.14 cm^2), and a glassy carbon working electrode (CHI104, 3 mm diameter). Solutions were prepared using dry and degassed DMA at a concentration of 2 mM metal complex. The $[NBu_4]PF_6$ electrolyte concentration was 0.1 M. Voltammograms were referenced to the $Cp_2Fe^{+/0}$ couple using ferrocene as an internal standard. Solution phase effective magnetic moments were determined using the Evans method. A dried solid analyte sample was dissolved in 0.800 mL of 9:1 v/v $CH_2Cl_2/PhCF_3$. This solution was added to a borosilicate NMR tube, along with a flame-sealed glass capillary containing a 4:1 v/v $CH_2Cl_2/PhCF_3$ internal standard. Elemental analyses were performed on a PerkinElmer 2400 Series II Analyzer at the CENTC Elemental Analysis Facility, University of Rochester.

S2. Syntheses and Characterization.

Synthesis of anhydrous iron(II) acetate. A 1 L three-necked flask was charged with acetic acid (150 mL, 2.62 mol) and acetic anhydride (40.6 mL, 0.43 mol). This mixture was then sparged with N_2 for 1 h. The solution was then stirred vigorously, and the flask was charged with iron powder (20 g, 0.36 mol) against a purge of N_2. The reaction was heated to reflux under an N_2 atmosphere for 48 h. The reaction was then cooled and all liquids were removed via cannula filtration. The solid remaining in the flask was dried *in vacuo* using a secondary solvent trap. Once the pressure reached 50 mTorr, the flask was then brought into the glovebox. The solids were then washed with 75 mL $E_{t2}O$ three times, then 75 mL THF three times, and then 75 mL Et_2O again three times. The solids were then dried under high vacuum at 100 ºC for 8 h. (*Note: drying large quantities of the product at this step will make it difficult to remove all of the acetic anhydride/acetic acid, and thus it is recommended to dry small batches in 20 mL scintillation vials*). The resulting anhydrous $Fe(OAc)_2$ was colorless or off-white in color, and was stored in the glovebox. Calculated elemental analysis for $C_4H_6O_4Fe$: C, 27.62%; H, 3.48%; N, 0%. Found: C, 27.96%; H, 3.31%; N, 0%. X-band EPR measurements confirm the valence purity of these samples.

Synthesis of 2,4,6-tri-*tert*-butylphenol-d_1 (TTBP-d_1). In the glovebox, a 20 mL scintillation vial was charged with 2,4,6-tri-*tert*-butylphenol (500 mg, 1.91 mmol) and 15 mL of anhydrous degassed THF. KH (91.7 mg, 2.29 mmol) was then added to the reaction portion wise and allowed to stir for 1 h. The reaction was then brought out of the glove box, D_2O (689 uL, 38.1 mmol) was added, and the reaction was stirred for 30 min. The reaction was then extracted with 3 x 100 mL Et_2O. The organic fractions were then dried in *vacuo* at ambient temperature. The solid was then brought into the glove box and dissolved in 3 mL anhydrous degassed Et_2O and filtered through Celite. The filtrate was then dried in *vacuo* at ambient temperature for 5 hours to yield a colorless solid. Deuterium incorporation was determined to be 96 % via ^{1}H NMR. Yield: 0.462 g, 1.91 mmol, 92%. The ^{1}H NMR spectrum of this compound matched that reported previously.[6]

Synthesis of [K(Crypt)][1]. In the glovebox, a scintillation vial was charged with potassium hexamethyldisilylamide (1.38 g, 6.93 mmol, 4.5 equiv.) and DMA (18 mL). After full dissolution, solid $Fe(OAc)_2$ (0.402 g, 2.31 mmol, 1.5 equiv.) was added over several minutes with vigorous stirring, which was continued for 30 min after addition was complete. The resulting suspension was vacuum filtered through a fine fritted funnel. The colorless filtrate was then added dropwise into a 20 mL scintillation vial charged with solid H_3L^{OCH2O} (1.00 g, 1.54 mmol). This reaction mixture was then stirred for 8 h at 70 °C. After cooling, the suspension was added slowly into MeCN (200 mL) while stirring vigorously. After stirring for 30 min the suspension was vacuum filtered using a medium fritted funnel. The colorless precipitate was washed with MeCN (5 mL) five times and allowed to dry under vacuum for 1 h. The precipitate was added into a scintillation vial containing 2.2.2-cryptand (696 mg, 1.85 mmol, 1.2 equiv.) and DCM (10 mL). After stirring for 15 min the suspension was vacuum filtered through a fine fritted funnel and washed with DCM (1 mL) three times. The filtrate was then dried under

vacuum while heating to 40 ºC to give a colorless solid, which was then triturated with THF (10 mL) for 8 h. The resulting suspension was vacuum filtered using a fine fritted funnel and washed with THF (1 mL) three times. The filtrate was then dried *in vacuo* to yield **[K(Crypt)][1]** as an analytically pure colorless solid. Yield: 0.700 g, 1.54 mmol, 43%. ^{1}H NMR (500 MHz, MeCN-d_3): δ = 28.53, 17.07, 14.68, 12.20, 3.38-3.33 (crypt-222), 2.38-2.34 (crypt-222), 0.68 ppm. μ_{eff} = 5.6 μ_B (Evans method, ^{19}F NMR, CH_2Cl_2/$PhCF_3$, 23 °C). Calculated elemental analysis for $C_{48}H_{63}N_6O_{15}FeK$: C, 54.44%; H, 6.00%; N, 7.94%. Found: C, 54.42%; H, 5.95%; N, 7.66%. Single crystals suitable for X-ray diffraction were grown by dissolving the product in DMA and then layering with Et_2O in the glovebox.

Synthesis of [K(Crypt)][2]. In the glovebox, a scintillation vial was charged with **[K(Crypt)][1]** (0.170 g, 0.161 mmol), iodosylbenzene (353 mg, 1.61 mmol, 10 equiv.) and MeCN (10 mL). The reaction was stirred in the freezer at –36 ºC for 3 days. After warming to ambient temperature, the suspension was vacuum filtered through a fine fritted funnel and the filter cake was washed with MeCN (1 mL) three times. The orange filtrate was then dried *in vacuo*. The resulting solid was triturated with THF (10 mL) for 1 h. The suspension was then vacuum filtered using a fine fritted funnel and washed with THF (1 mL) 3 times. The orange solid was then dried *in vacuo* at 70 °C for 6 hours to yield **[K(Crypt)][2]** as an analytically pure orange powder. Yield: 0.117 g, 0.109 mmol, 68%. ^{1}H NMR (500 MHz, MeCN-d_3): δ = 28.59, 17.03, 14.67, 12.18, 3.42-3.36 (crypt-222), 2.42-2.37 (crypt-222), 0.69, -2.28 ppm. μ_{eff} = 5.4 μ_B (Evans method, ^{19}F NMR, CH_2Cl_2/$PhCF_3$, 23 °C). Calculated elemental analysis for $C_{48}H_{63}N_6O_{16}FeK$: C, 53.63%; H, 5.91%; N, 7.82%. Found: C, 53.41%; H, 6.15%; N, 7.67%. ESI-MS (MeCN): *m/z* calculated for **[2]**$^-$: 659.11 Found: 659.59. Single crystals suitable for X-ray diffraction were grown by dissolving the product in DMA and then layering with Et_2O in the glovebox. Synthesis of ^{18}O-labelled **[K(Crypt)][2]** utilized iodosylbenzene-^{18}O using an otherwise identical procedure.

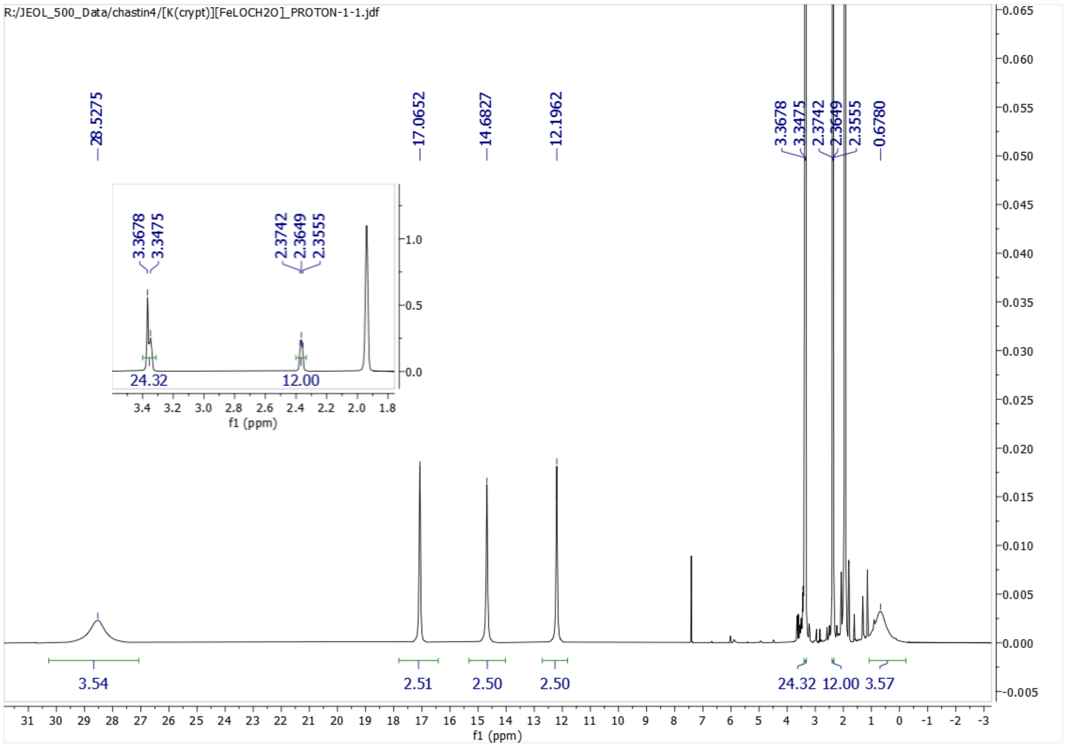

Figure S1. ^{1}H NMR (500 MHz) spectrum of **[K(Crypt)][1]** in CD_3CN at 23 ºC.

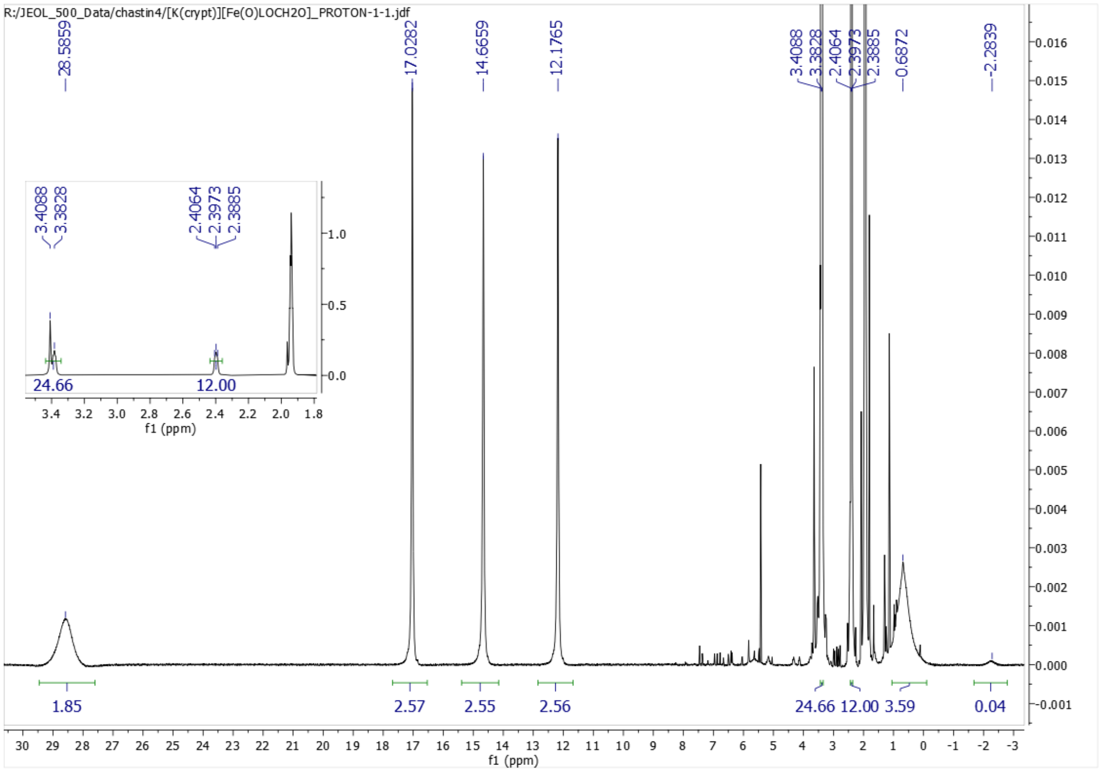

Figure S2. ^{1}H NMR (500 MHz) spectrum of **[K(Crypt)][2]** in CD_3CN at 23 ºC.

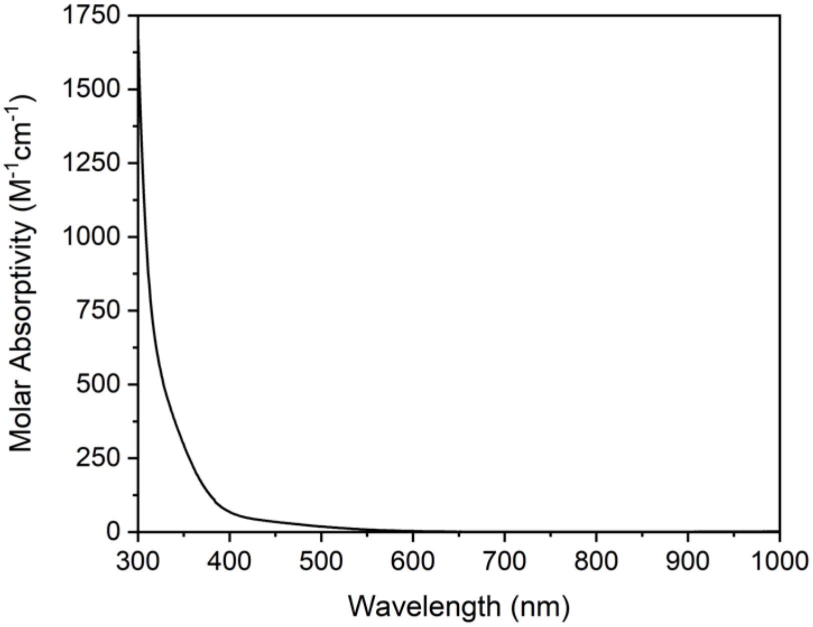

Figure S3. Electronic spectrum of **[K(Crypt)][1]** in MeCN at 23 ºC.

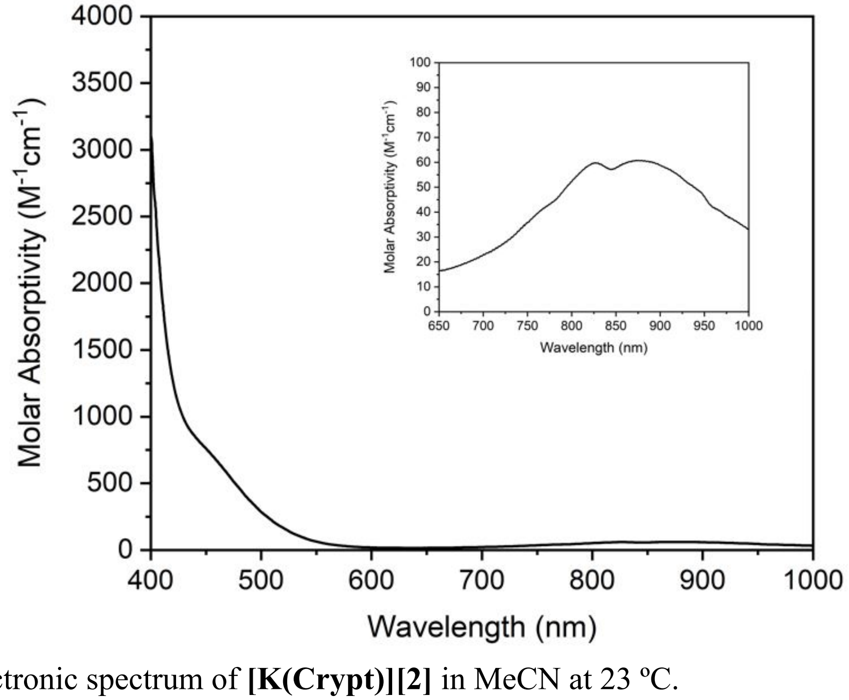

Figure S4. Electronic spectrum of **[K(Crypt)][2]** in MeCN at 23 ºC.

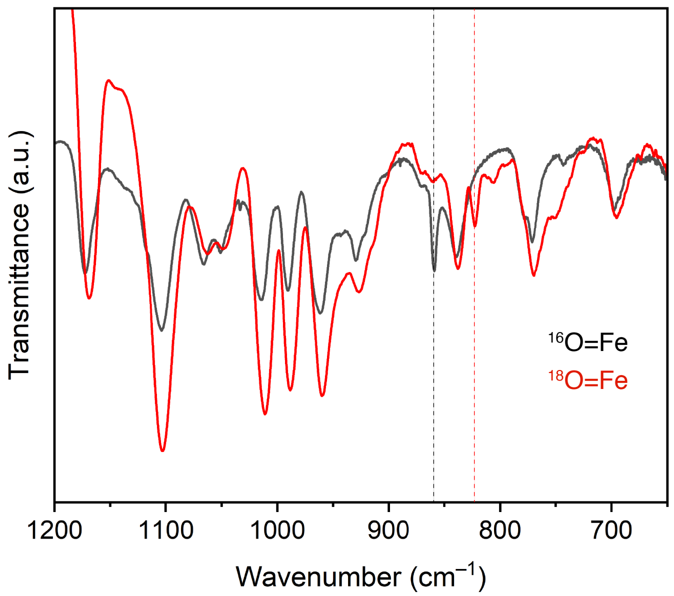

Figure S5. Attenuated total reflectance infrared spectra of **[K[Crypt]][2]**-^{16}O (black) and **[K[Crypt]][2]**-^{18}O (red). The dashed vertical lines denote the positions of the features ascribed to υ(Fe=O).

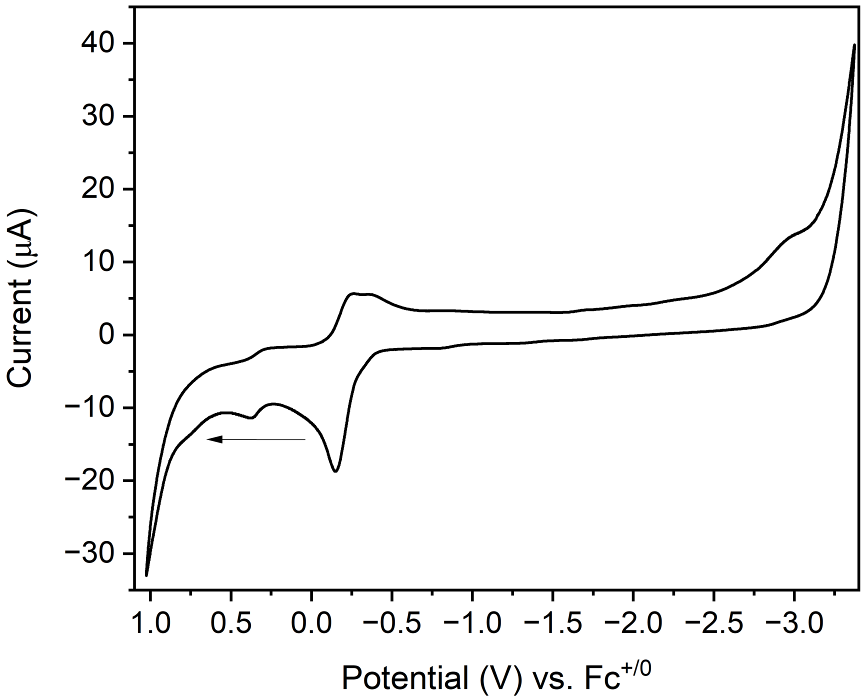

Figure S6. Full window cyclic voltammogram of [**2**]$^{-}$ in DMA (scan rate = 0.1 V/s).

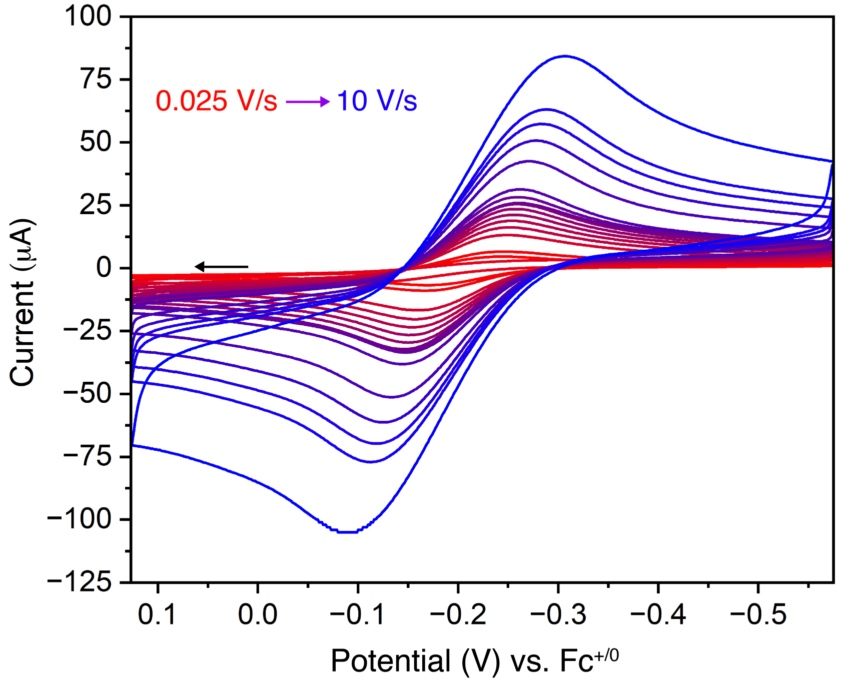

Figure S7. Cyclic voltammogram of [**2**]$^{-}$ in DMA focusing on the quasi-reversible feature assigned as $Fe^{IV/III}$. Scan rates shown are (V/s): 0.025; 0.05; 0.1; 0.2; 0.3; 0.4; 0.5; 0.6; 0.7; 0.8; 0.9; 1; 2; 3; 4; 5; 10.

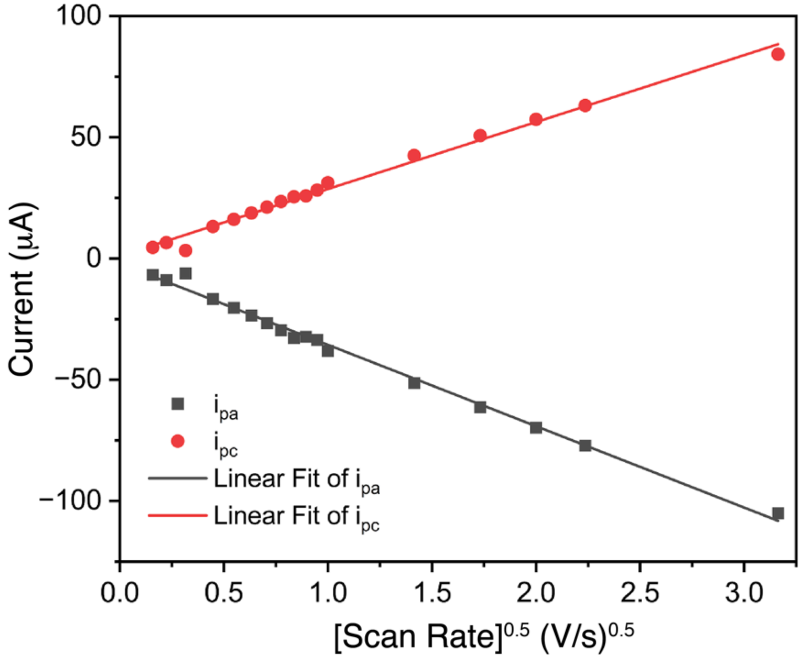

Figure S8. Relationship between square root of the scan rate and the peak current for the $Fe^{IV/III}$ couple. Peak current during the anodic sweep is denoted as i_{pa}, and peak current during the cathodic sweep as i_{pc}. The lines represent least squares linear regressions.

Synthesis of [K(18-crown-6)][OH]. In the glovebox, a scintillation vial was charged with finely ground KOH (2.12 g, 37.8 mmol, 1.0 eq), 18-crown-6 (10.0 g, 37.8 mmol, 1.0 eq), and 2 mL of MeOH. The slurry was stirred at 60 ºC for 1 h before filtering through Celite. The filtrate was dried *in vacuo* and crystals were grown from CH_2Cl_2 in the freezer.

Independent Generation of [K(18-crown-6)][Fe(OH)L^{OCH2O}]. This procedure utilized the [K(18-crown-6)]$^+$ salt of the ferrous starting material [**1**]$^-$, which was generated using the synthetic procedure for [K(Crypt)][**1**] by replacing [2.2.2]cryptand with an equimolar quantity of 18-crown-6.

In situ production of FeL^{OCH2O}: As reported previously,[6] a scintillation vial was charged with FcOTf (8.9 mg, 26 μmol, 1.0 eq) and 0.5 mL of CH_2Cl_2 in the glovebox. The solution was added to a scintillation vial containing solid [K(18-crown-6)][FeL^{OCH2O}] (25 mg, 26 μmol, 1.0 eq) and a stir bar, and stirred at 23 ºC for 5 min. To the resulting brown solution was added 20 mL of pentane to precipitate the crude complex before it was filtered through a fine porosity fritted funnel.

Hydroxide addition: The dried filter cake was added to a scintillation vial containing [K(18-crown-6)][OH] and 2 mL CH_2Cl_2 and let stir for 20 min. To the resulting yellow solution was added 20 mL Et_2O and the suspension was vacuum filtered using a fine fritted funnel. The yellow solid was then dried in vacuo at 70 °C for several hours. The resulting solid was analyzed by EPR (Figure 3) and IR (Figure S9) spectroscopies, as well as high-resolution mass spectrometry (Figure S10). Repeated attempts to access single crystals and analytically pure material have been unsuccessful.

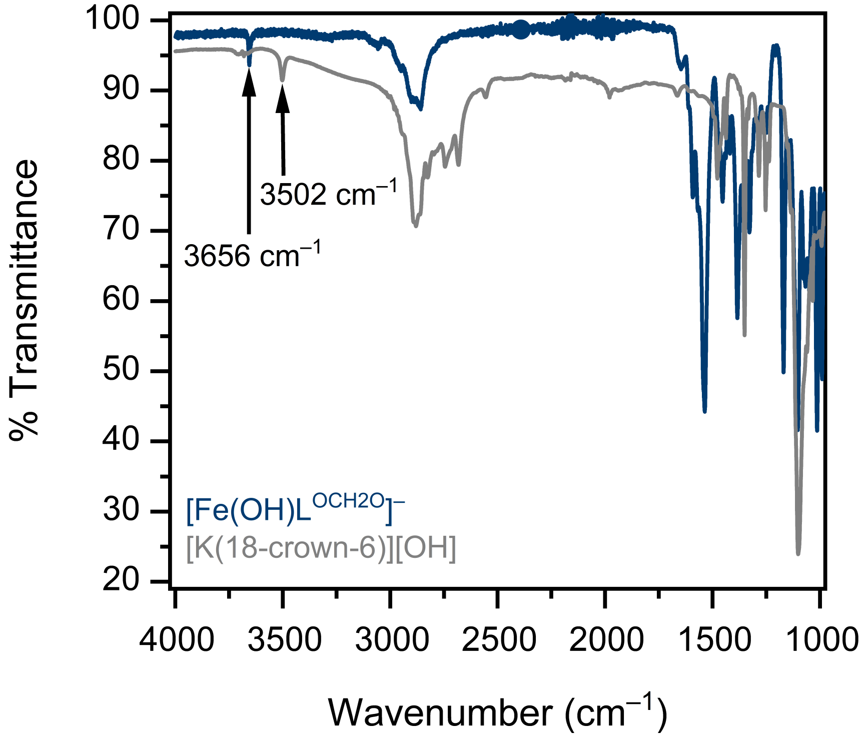

Figure S9. Transmission infrared spectra of independently generated [Fe(OH)L^{OCH2O}]$^-$ (blue) and [K(18-crown-6)][OH] (grey).

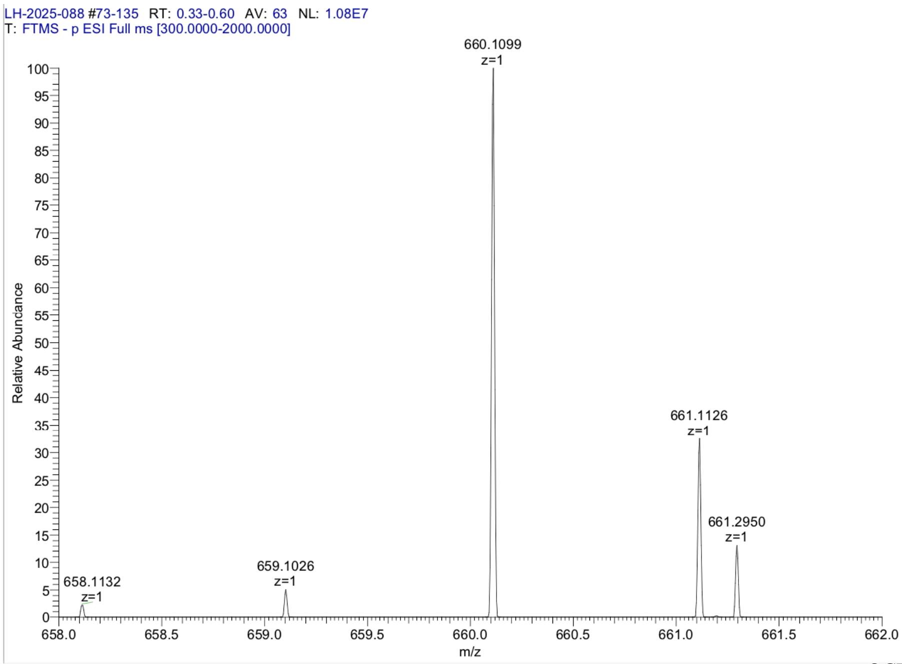

Figure S10. High-resolution mass spectra of independently generated [Fe(OH)L^{OCH2O}]$^{-}$ obtained as a dilute MeCN solution. The most abundant peak is predicted to occur at 660.1155 *m/z* (Δ = 8 ppm), with the second-most abundant peak at 661.1189 *m/z* (Δ = 10 ppm; predicted relative intensity of 32%). The deviation between experimental and calculated values are within the specifications of the instrument (± 10 ppm).

S3. Kinetic Measurements of Self-Decay and Attempted Reactions with CHD, DHA, and Potential O-atom Acceptors.

Experimental Procedure for Monitoring the Thermal Decay of [2]$^{-}$ in acetonitrile. Inside of the glovebox, a cuvette was charged with a 0.5 mM solution of [**2**]$^{-}$ in CH_3CN. The cuvette was sealed, removed from the glovebox, and placed into an electronic spectrometer with the sample cell heated to 70 ºC.

Experimental Procedure for Reactions of [2]$^{-}$ with PMe_3, Styrene, CHD and DHA. Inside the glovebox, a 20 mL scintillation vial was charged with 400 μL of a 10 mM stock solution of **[2]**$^{-}$ in MeCN-*d*$_3$**,** 40 mmol of substrate, and 400 μL of MeCN-*d*$_3$. After full dissolution of the substrate the solution was transferred into a J-Young NMR tube and sealed. It was then brought outside the glovebox and reacted at 70 °C for 12 h by heating in an oil bath.

Experimental Procedure for Reactions of [2]$^{-}$ with C_2H_4. Inside the glovebox, a J-young tube was charged with 400 μL of a 10 mM stock solution of **[2]**$^{-}$ in MeCN-*d*$_3$ and diluted with 400 μL of MeCN-*d*$_3$. The tube was then sealed and brought out of the glovebox and connected to the Schlenk line. The tube was degassed via three freeze-pump-thaw cycles. The tube was then backfilled with 1 bar C_2H_4, sealed and shaken vigorously. The tube was then heated at 70 °C for 12 h by heating in an oil bath.

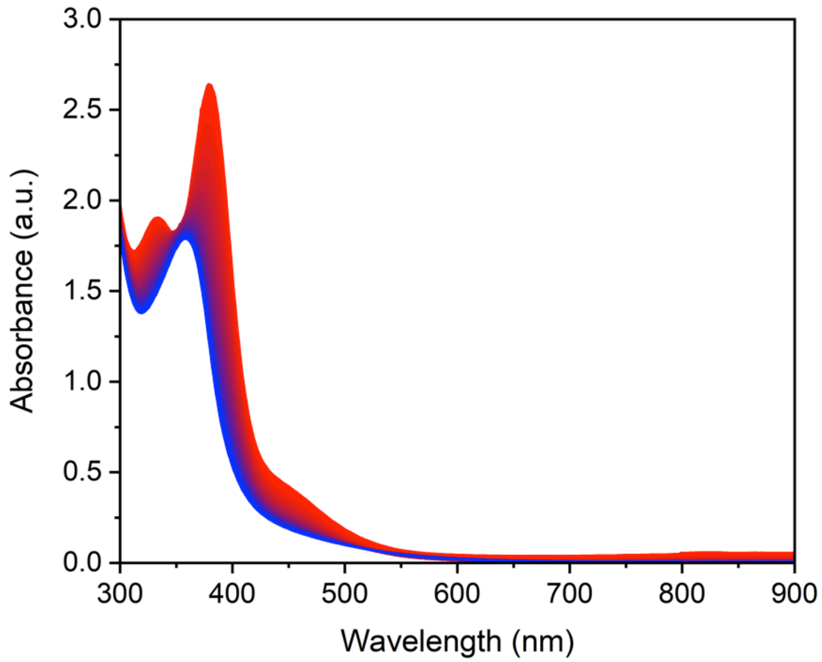

Figure S11. Thermal decay of [**2**]$^{-}$ (0.5 mM) at 70 ºC in acetonitrile as monitored by electronic spectroscopy. The time dimension is denoted with a red to blue gradient. Spectra were acquired over 50 h at 5 min intervals.

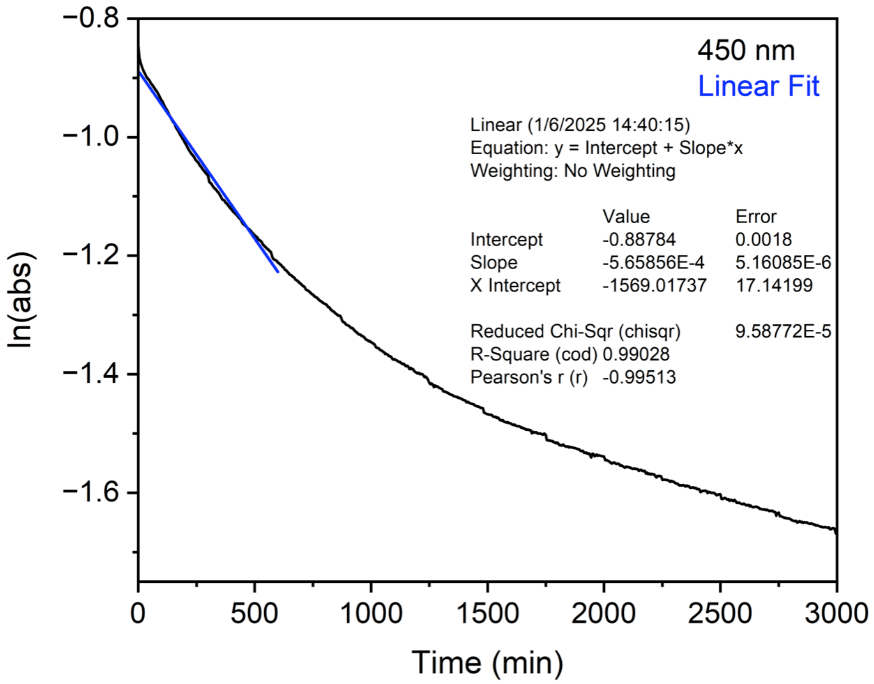

Figure S12. Thermal decay of [**2**]$^{-}$ at 70 ºC in acetonitrile as monitored by the absorbance at 450 nm in the electronic spectrum. Least-squares linear regression (using data from 0-600 min) is shown with a blue line. Deviation from linearity at longer times is due to generation of one (or more) byproducts with significant absorption at this wavelength. The linear regression yields a first-order rate constant of $5.6 \cdot 10^{-4}$ min^{-1}, which corresponds to a half-life of 21 h.

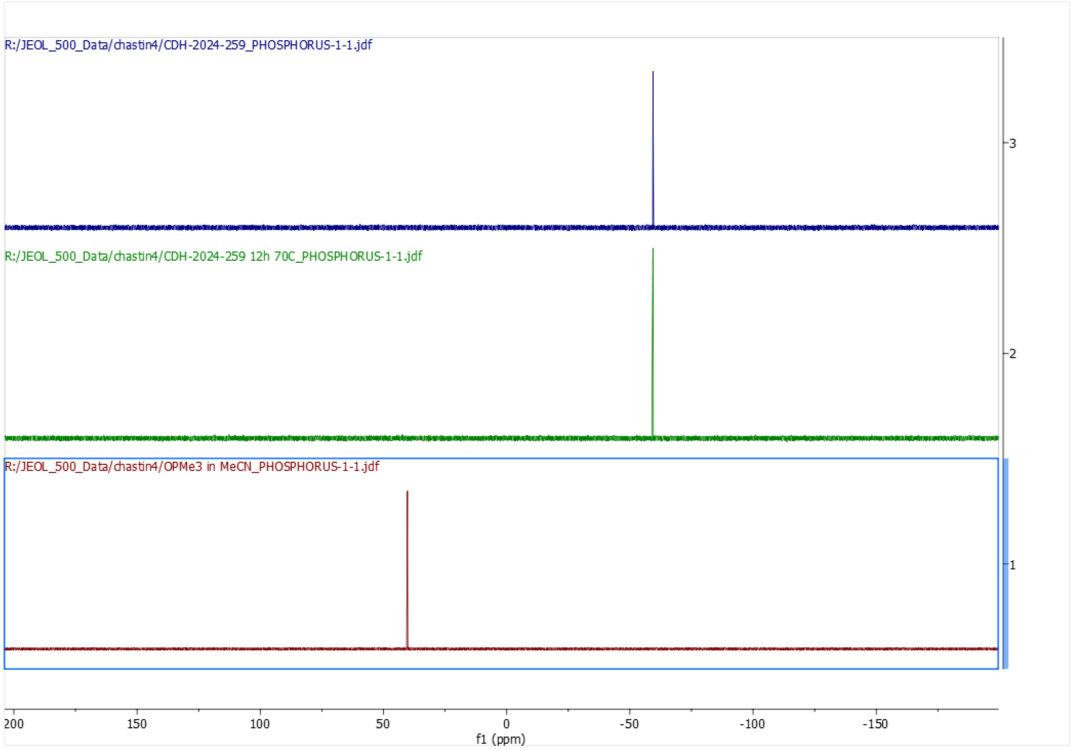

Figure S13. $^{31}P\{^{1}H\}$ NMR spectra (202 MHz, 23 ºC) of the reaction of [**2**]$^{-}$ (5 mM) and PMe_3 (50 mM) in CD_3CN. The top spectrum was taken immediately after mixing, while the middle spectrum was taken after the reaction mixture in the J-Young NMR tube was heated to 70 ºC for 12 h. The peak at –59.37 ppm corresponds to PMe_3. Trimethylphosphine oxide ($OPPMe_3$), the expected product of O-atom transfer, would give rise to a singlet at 40.28 ppm in CD_3CN as shown in the authentic reference spectrum (bottom).

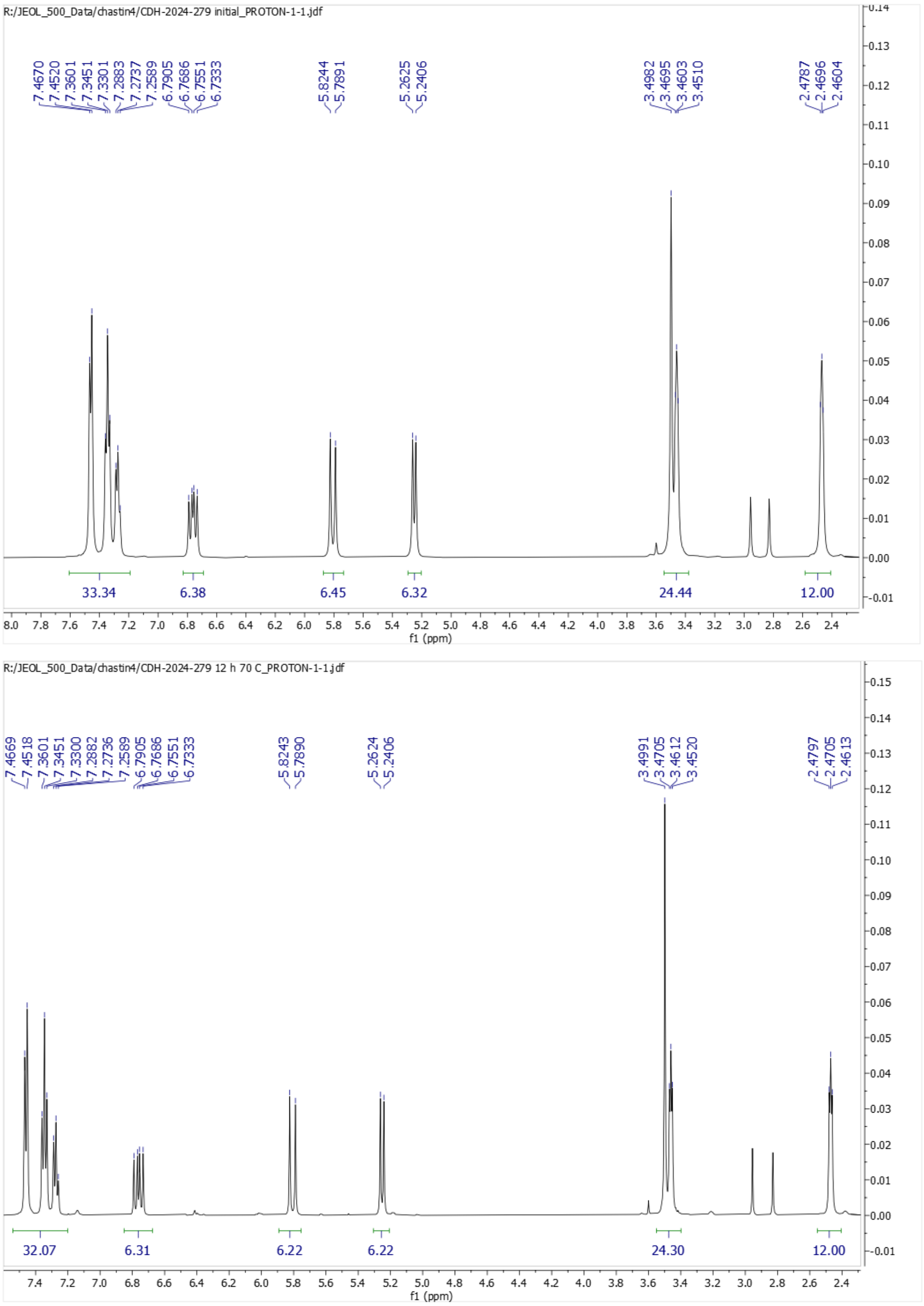

Figure S14. ^{1}H NMR spectra (500 MHz, 23 ºC) of the reaction of [**2**]$^-$ (5 mM) and styrene (30 mM) in CD_3CN. The top spectrum corresponds to t = 0, while the bottom spectrum was taken after the J-Young tube was heated to 70 ºC for 12 h. The peaks observed in the aromatic portion of the spectrum before and after heating correspond only to styrene. The peaks centered at 2.47, 3.45, and 3.47 ppm correspond to $[K(Crypt)]^+$ and serve as *de facto* internal standards to assess the quantity of substrate with respect to [**2**]$^-$ that was present at t = 0.

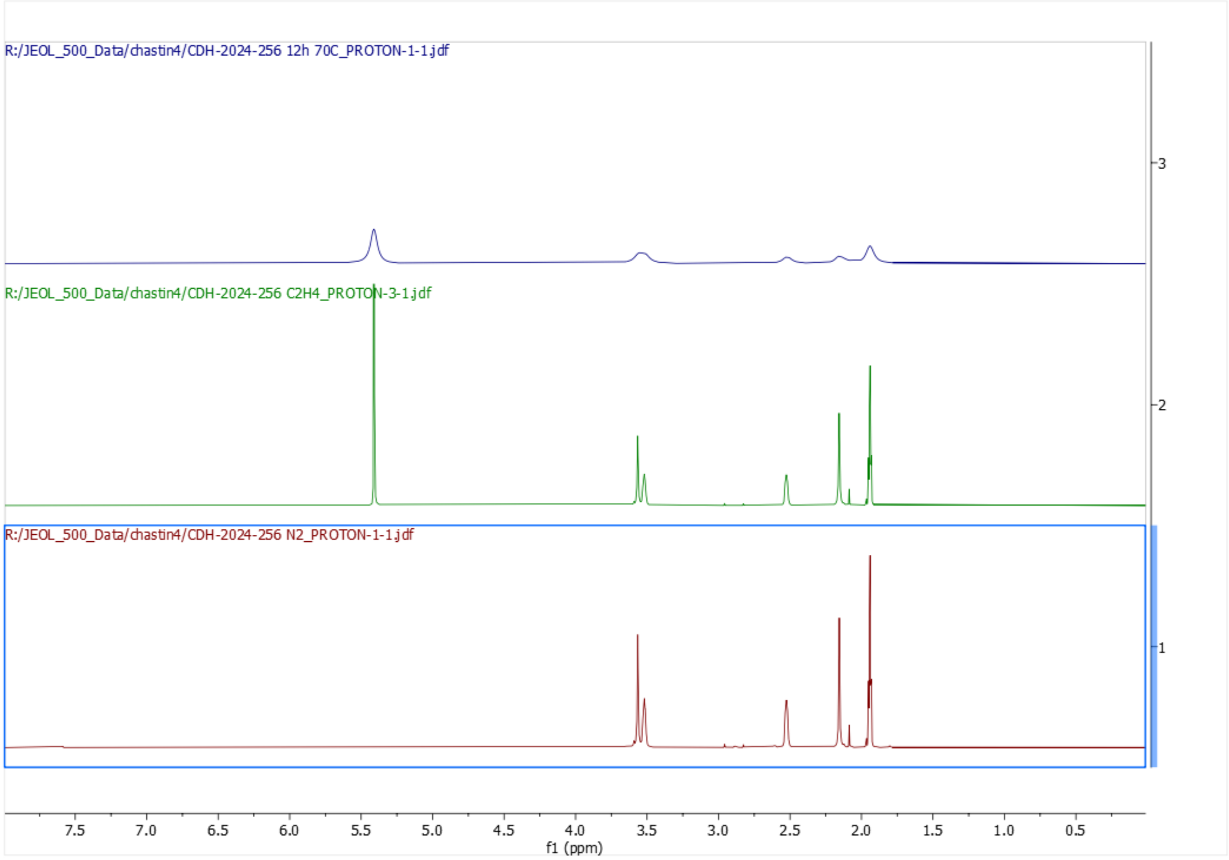

Figure S15. ^{1}H NMR spectra (500 MHz, 23 ºC) of the reaction of [**2**]$^{-}$ (5 mM) and ethylene (1 bar) in CD_3CN. The bottom spectrum corresponds to [**2**]$^{-}$ only, the middle spectrum corresponds to [**2**]$^{-}$ and ethylene prior to heating, and the top spectrum following heating the J-Young tube to 70 ºC for 12 h. No new peaks in the diamagnetic portion of the spectrum are evident. Zoom-ins of the spectra under ethylene are shown below.

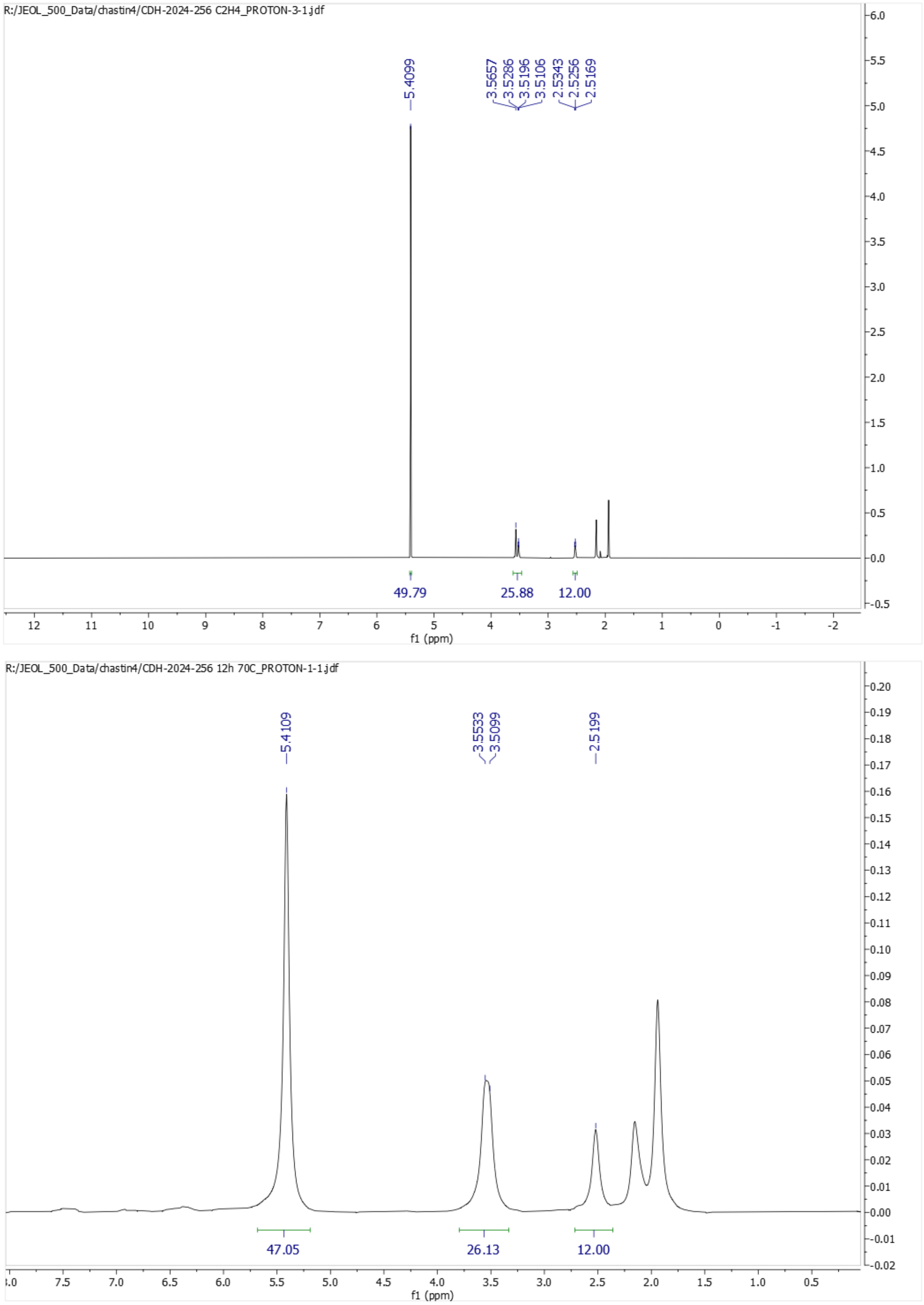

Figure S16. ^{1}H NMR spectra (500 MHz, 23 ºC) of the reaction of [**2**]$^-$ (5 mM) and ethylene (1 bar) in CD_3CN. The top spectrum was acquired immediately after ethylene addition (corresponds to the green data in Figure S15). The bottom spectrum was acquired after heating at 70 ºC for 12 h (corresponds to the blue spectrum in Figure S15).

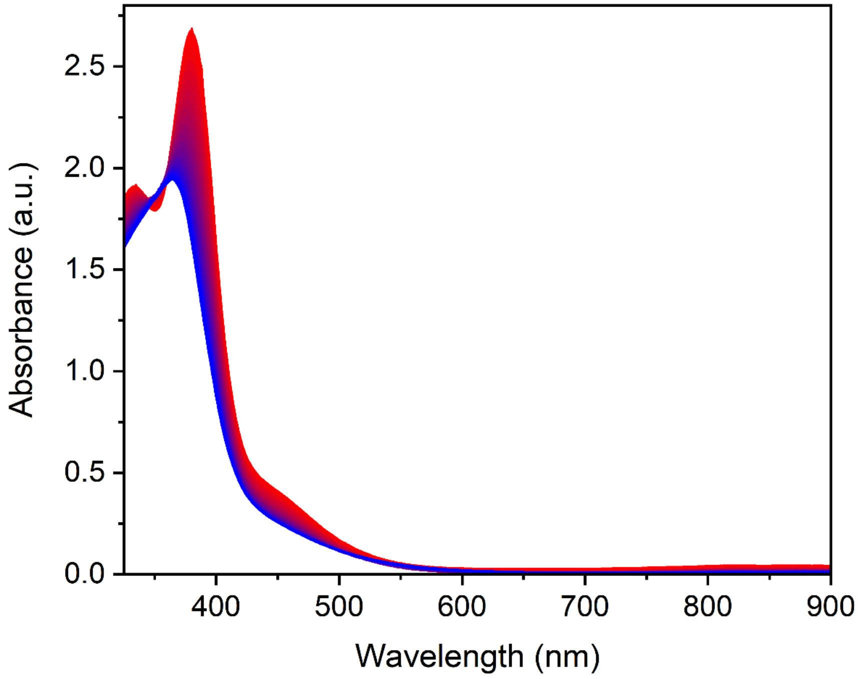

Figure S17. Thermal decay of [**2**]$^-$ (0.5 mM) at 70 ºC in acetonitrile in the presence of 10 eq. 1,4-cyclohexadiene (CHD) as monitored by electronic spectroscopy. The time dimension is denoted with a red to blue gradient. Spectra were acquired over 24 h at 5 min intervals.

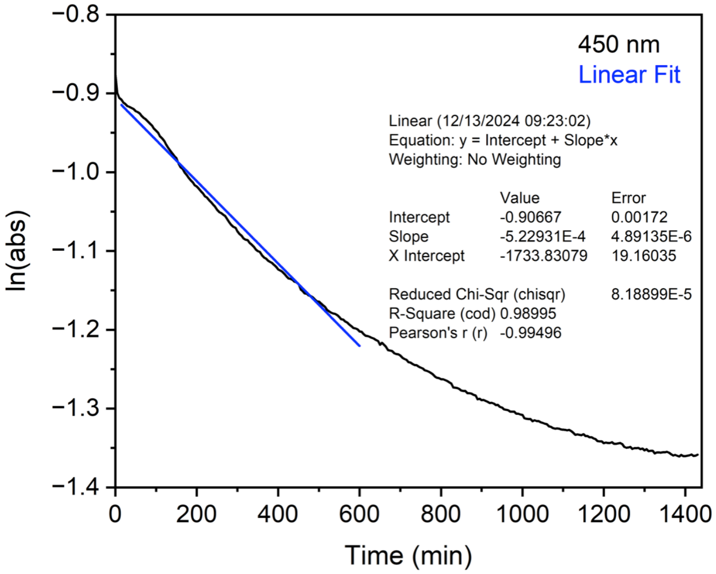

Figure S18. Thermal decay of [**2**]$^-$ at 70 ºC in acetonitrile in the presence of 10 equivalents of 1,4-cyclohexadiene (CHD) as monitored by the absorbance at 450 nm in the electronic spectrum. The resulting first order rate constant k_1 is 5.2 • 10^{-4} s^{-1} as determined from a linear regression (blue line) of the data acquired between 0 to 600 min.

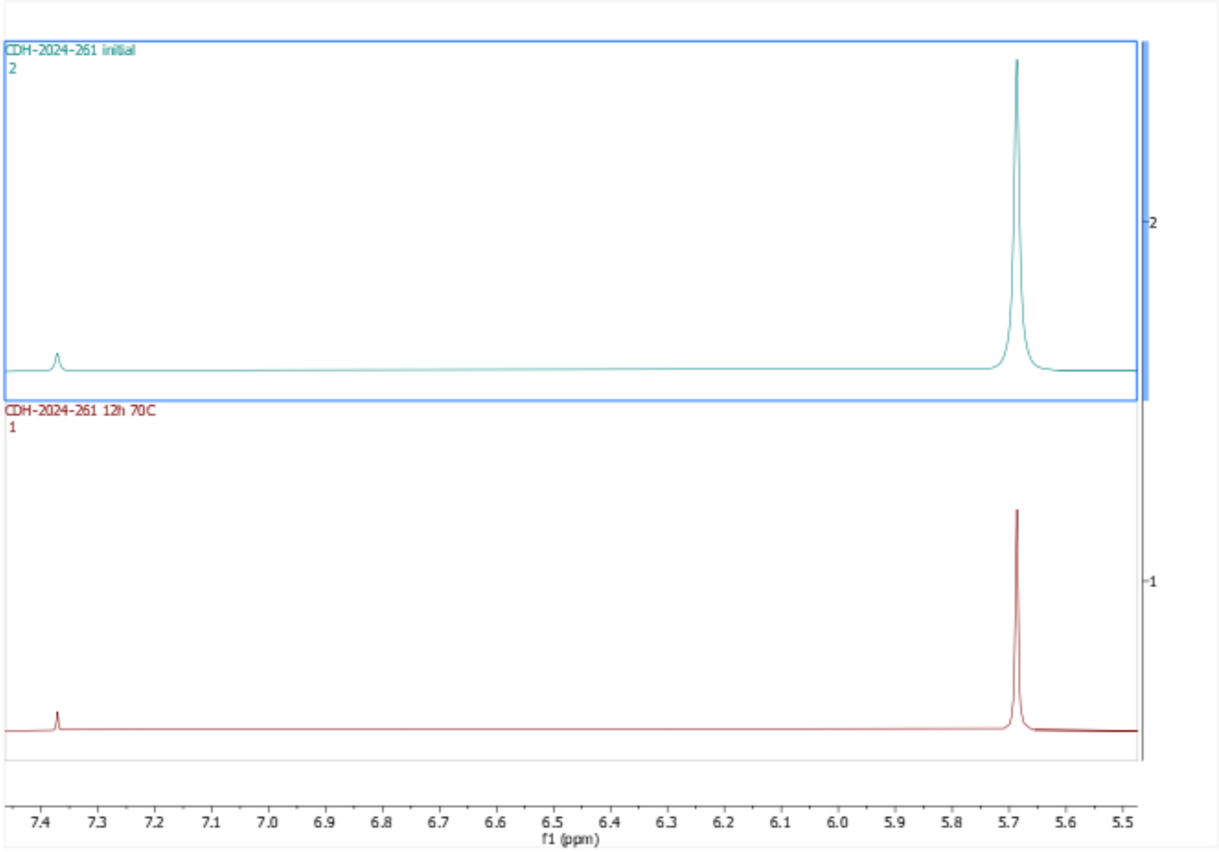

Figure S19. ^{1}H NMR spectra (500 MHz, 23 ºC) of the reaction of [**2**]$^-$ (5 mM) and CHD (50 mM) in CD_3CN. The top spectrum corresponds to t = 0, while the bottom spectrum was taken after the J-Young tube was heated to 70 ºC for 12 h. The peaks at 5.7 and 7.4 ppm correspond to CHD and benzene, respectively. The presence of a small amount of benzene in the top spectrum reflects its presence as an impurity in the CHD as confirmed from a control spectrum. As is evident, heating to 70 º for 12 h results in minimal change in the relative quantity of benzene present. Peak-picked and integrated spectra are shown below.

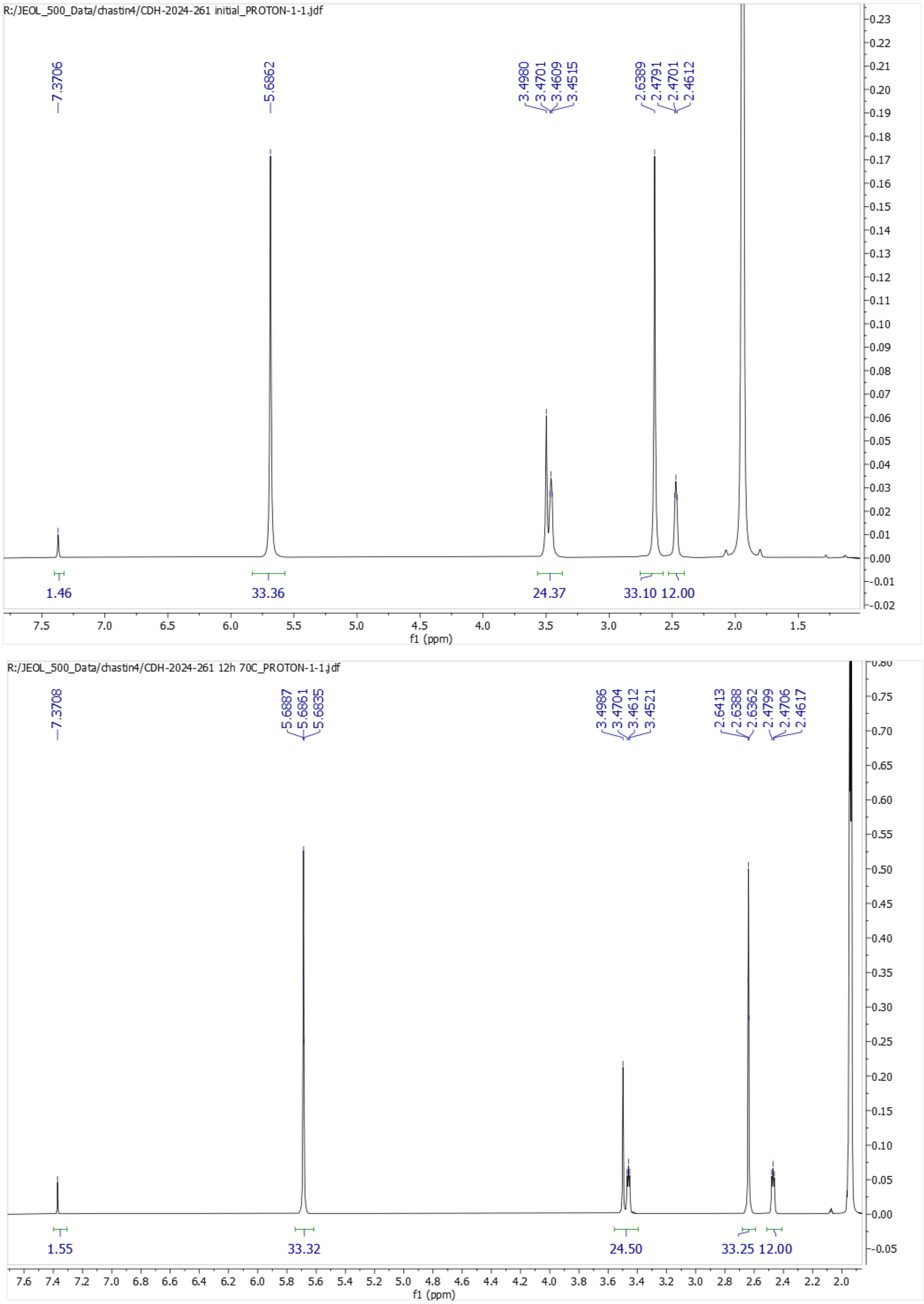

Figure S20. ^{1}H NMR spectra (500 MHz, 23 ºC) of the reaction of [**2**]$^{–}$ (5 mM) and CHD (50 mM) in CD_3CN. The top spectrum corresponds to t = 0 (green data in Figure S19), while the bottom spectrum was taken after the J-Young tube was heated to 70 ºC for 12 h (red data in Figure S19).

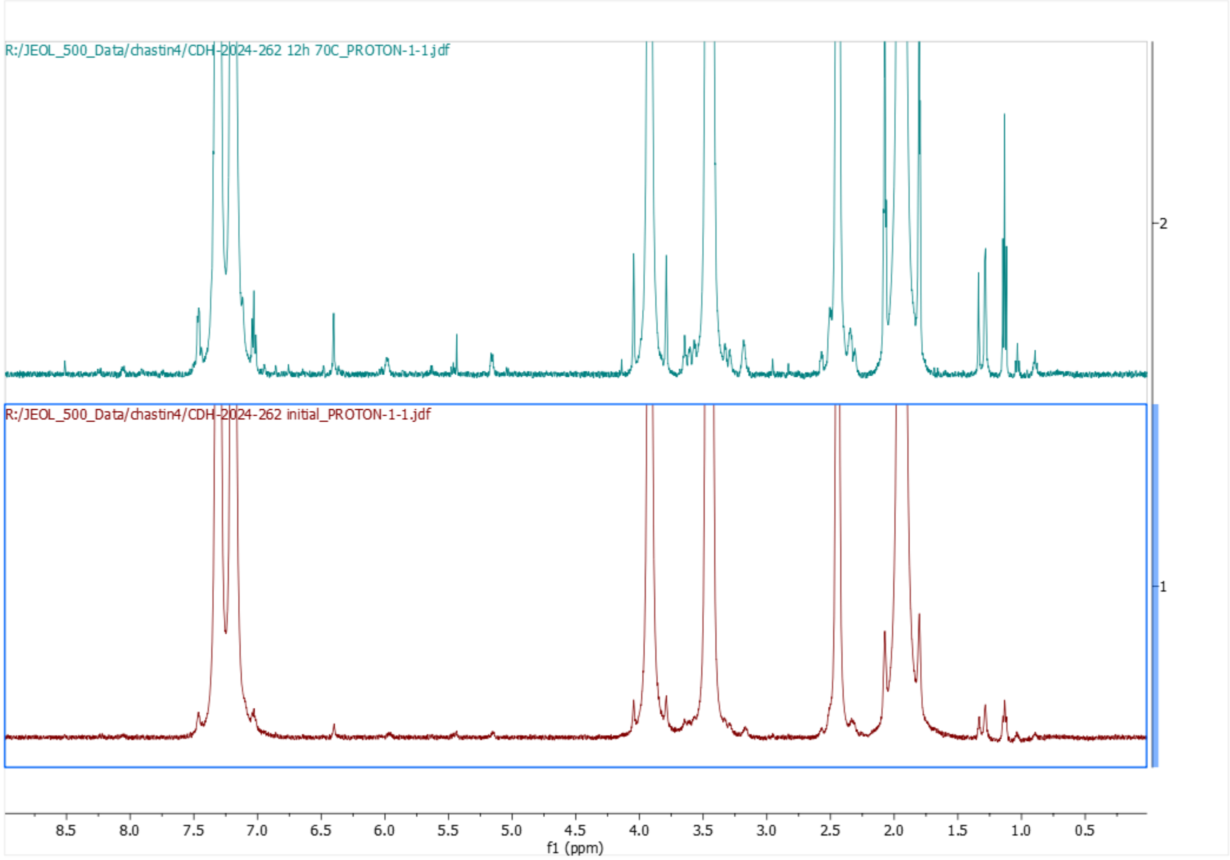

Figure S21. ^{1}H NMR spectra (500 MHz, 23 ºC) of the reaction of [**2**]$^{–}$ (5 mM) and DHA (50 mM) in CD_3CN. The top spectrum corresponds to *t* = 0, while the bottom spectrum was taken after the J-Young tube was heated to 70 ºC for 12 h. The peaks at 3.9, 7.2, and 7.3 ppm correspond to DHA starting material. Anthracene, the expected product of H-atom abstraction from DHA, gives rise to aromatic peaks between 8.52, 8.07-8.05 and 7.51-7.50 ppm in CD_3CN (see Figure S23). As is evident, heating to 70 º for 12 h results in no measurable production of anthracene. Peak-picked and integrated spectra are shown below.

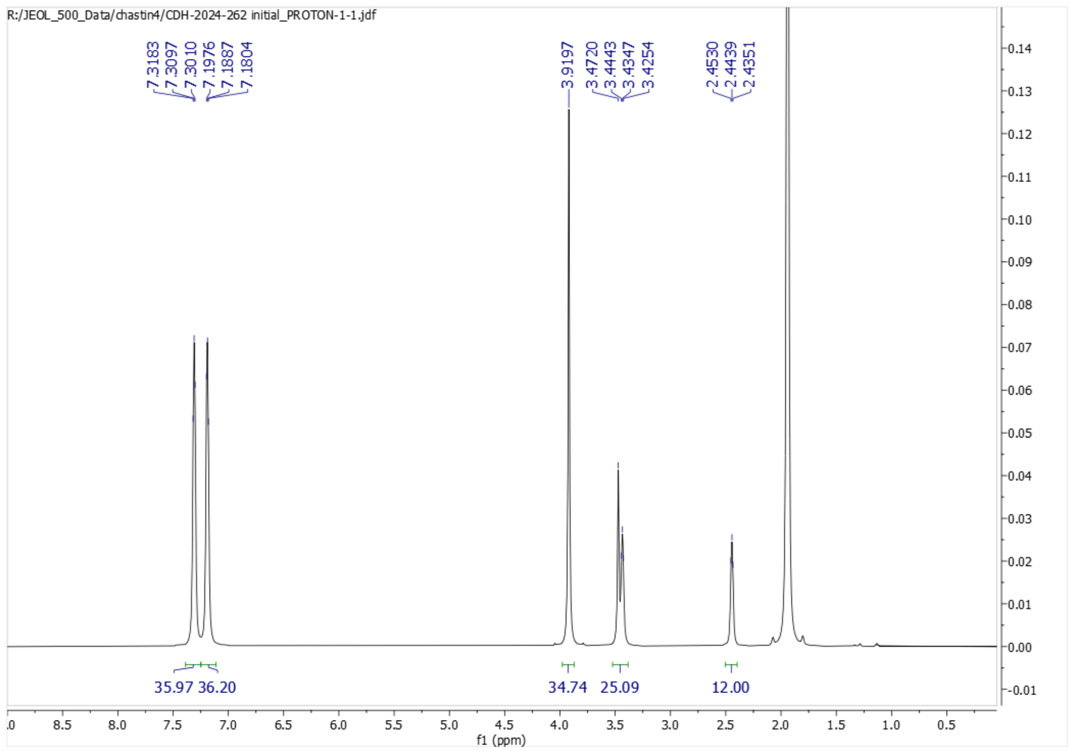

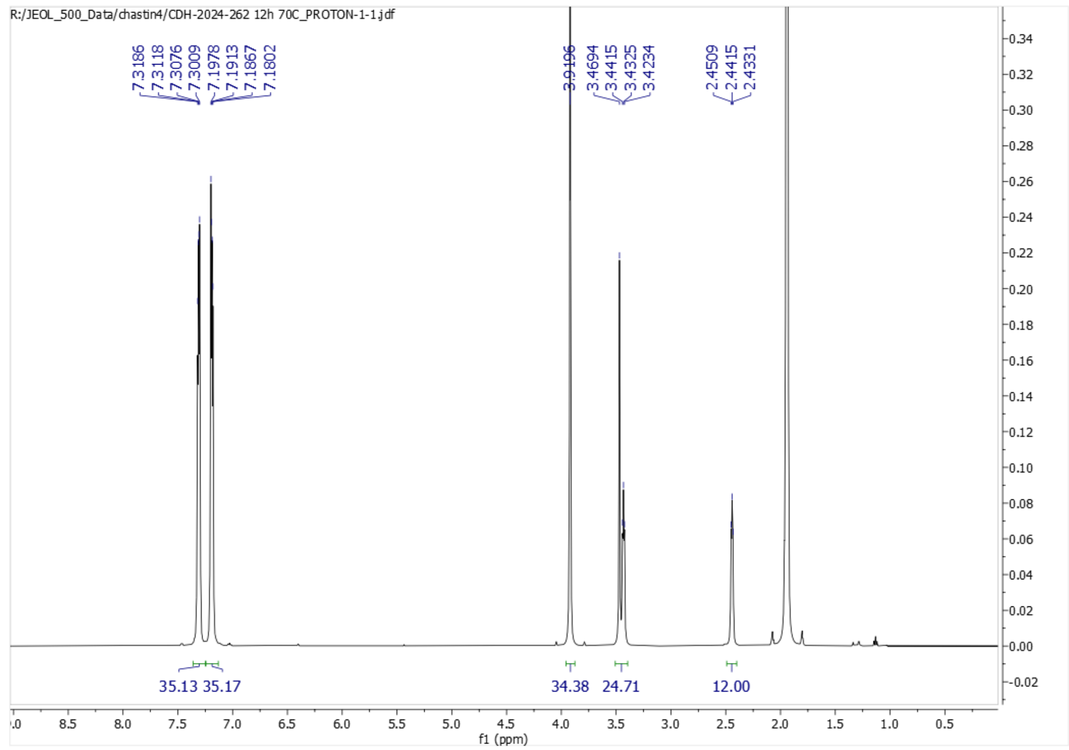

Figure S22. ^{1}H NMR spectra (500 MHz, 23 ºC) of the reaction of [**2**]$^-$ (5 mM) and DHA (50 mM) in CD_3CN. The top spectrum corresponds to t = 0 (green data in Figure S21), while the bottom spectrum was taken after the J-Young tube was heated to 70 ºC for 12 (red data in Figure S21).

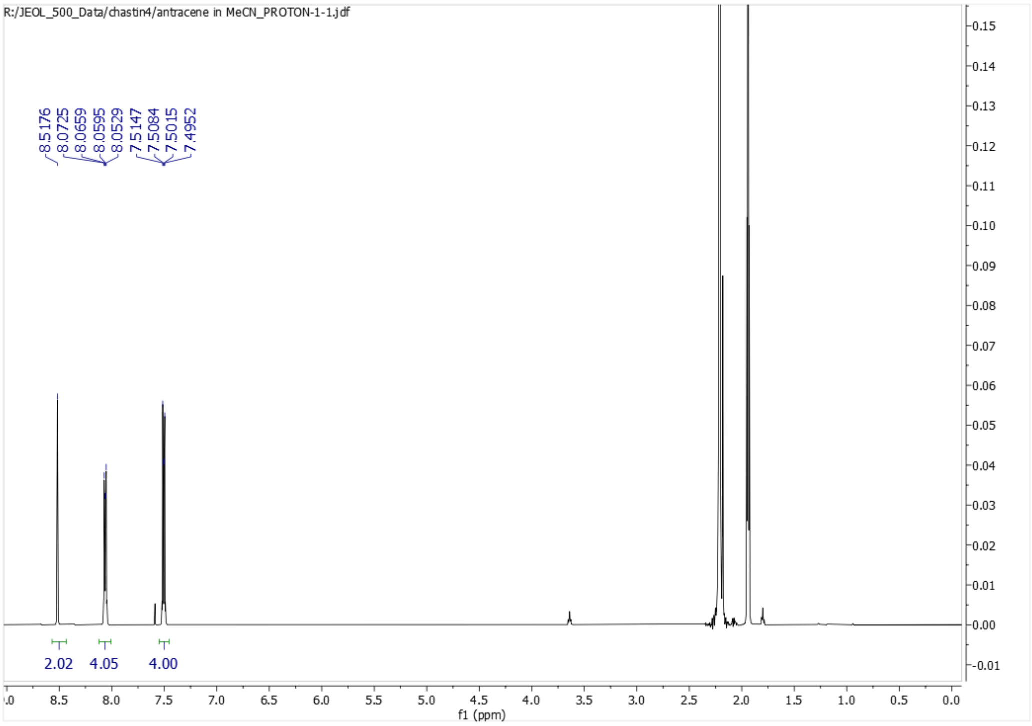

Figure S23. Authentic reference ^{1}H NMR spectrum (500 MHz, 23 ºC) of anthracene in MeCN-d_3.

S4. Reaction of [2]$^-$ with TTBP

Procedure. Inside the glovebox, a cuvette was charged with 200 μL of [**2**]$^-$ (10 mM stock solution in MeCN), 526 μL of TTBP (38 mM stock solution in MeCN) and 3.274 mL of anhydrous degassed MeCN. The cuvette was then brought out of the glovebox and placed into the spectrometer (sample sell pre-heated to 25 °C) within 2 min. Spectra were collected every 5 minutes for 24 h. After acquiring kinetics data, a small aliquot of the solution was removed, diluted in MeCN, and assayed by ESI-MS.

m/z expected for **[Fe(OH)L$^{\mathbf{OCH2O}}$]**$^-$: 660.11; Found: 660.08.

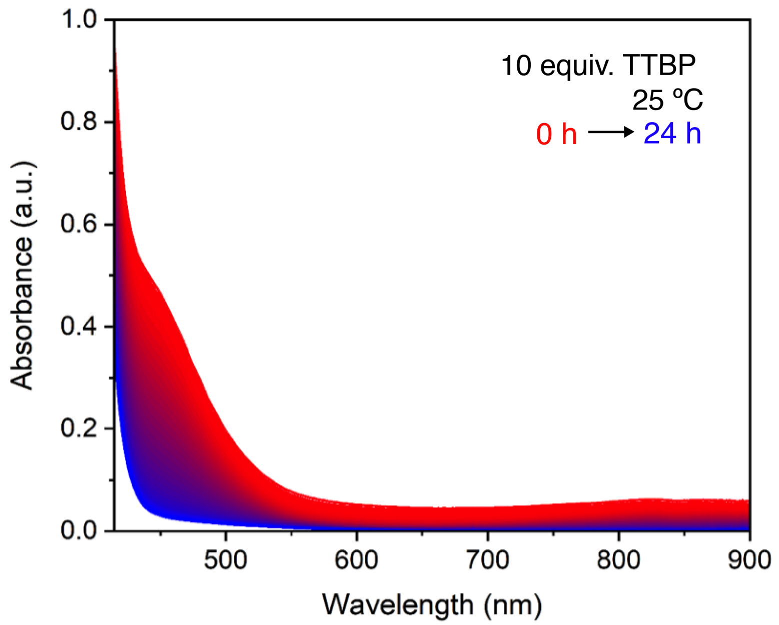

Figure S24. Reaction of [**2**]$^{-}$ with TTBP-d_0 (10 equivalents) at 25 ºC in acetonitrile as monitored by electronic spectroscopy. The time dimension is denoted with a red to blue gradient. Data points were acquired over 24 h at 5 min intervals.

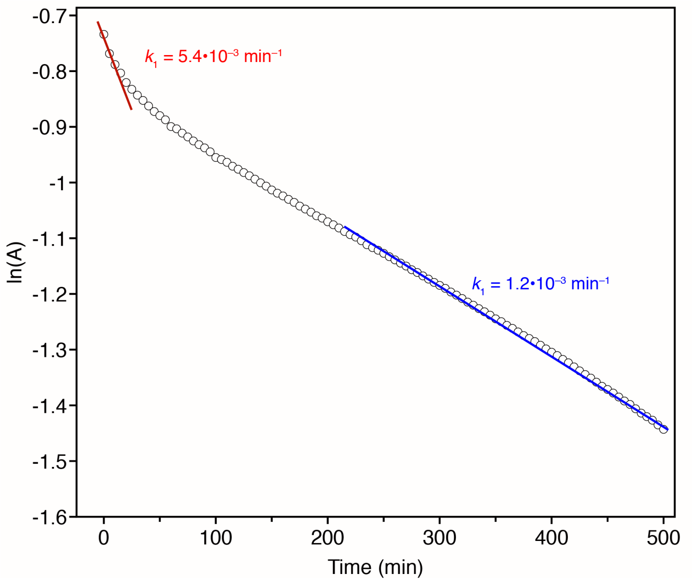

Figure S25. Reaction of [**2**]$^{-}$ with TTBP-d_0 (10 equivalents) at 25 ºC in acetonitrile as monitored by the absorbance at 450 nm in the electronic spectrum. Open black circles correspond to individual data points. The red line corresponds to a least-squares linear regression using the first three time points, and has a slope of $-5.4•10^{-3}$. The red line corresponds to a least-squares linear regression using time points between 200–500 min, and has a slope of $-1.2•10^{-3}$. These slopes represent the negative of the first-order rate constants for the given time points.

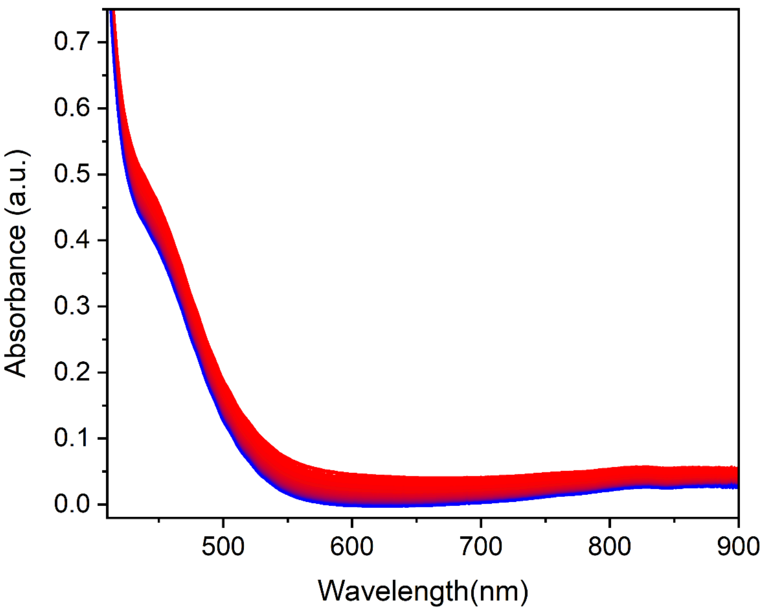

Figure S26. Reaction of [**2**]$^{-}$ with TTBP-d_1 (10 equivalents) at 25 ºC in acetonitrile as monitored by electronic spectroscopy. The time dimension is denoted with a red to blue gradient. Data points were acquired over 19 h at 5 min intervals.

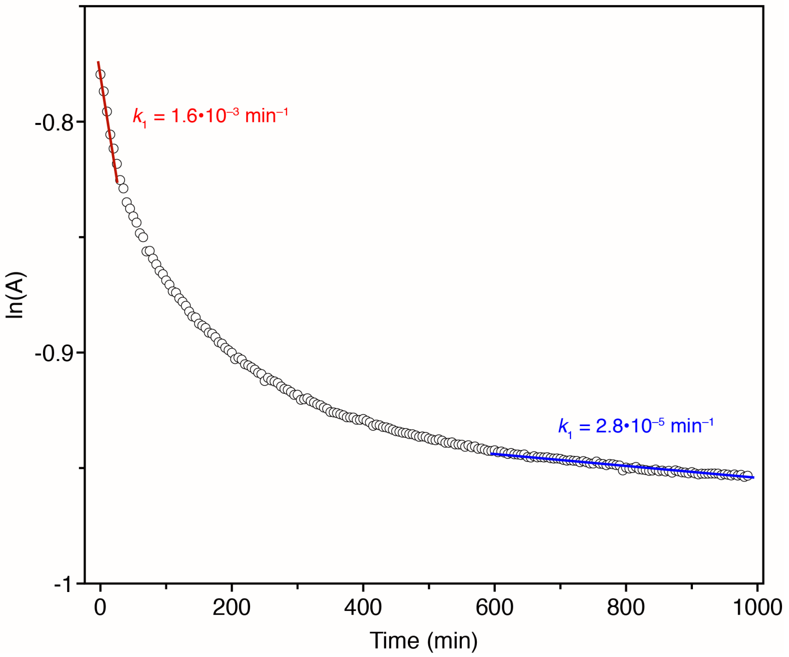

Figure S27. Reaction of [**2**]$^{-}$ with TTBP-d_1 (10 equivalents) at 25 ºC in acetonitrile as monitored by the absorbance at 450 nm in the electronic spectrum. Open black circles correspond to individual data points. The red line corresponds to a least-squares linear regression using the first three time points, and has a slope of $-1.6 \cdot 10^{-3}$. The red line corresponds to a least-squares linear regression using time points between 600–1000 min, and has a slope of $-2.8 \cdot 10^{-5}$. These slopes represent the negative of the first-order rate constants for the given time points.

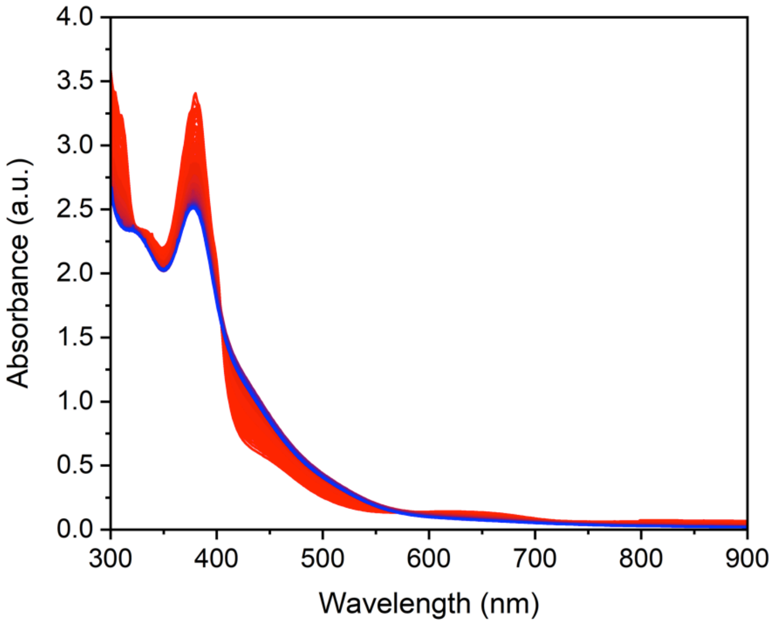

Figure S28. Reaction of [**2**]$^{-}$ with tri(2,4,6-*tert*-butyl)phenoxy radical (1 equivalent) at 25 ºC in acetonitrile as monitored by electronic spectroscopy. Spectra were acquired at 5 min intervals over 26 h. The time dimension is denoted with a red-to-blue gradient.

S5. Details of Density Functional Theory Calculations

S6.1. General Considerations. Calculations were carried out using the ORCA program package.[7] Geometry optimizations and single-point calculations were performed using the B3LYP hybrid DFT functional.[8,9] In all calculations, the functional was appended with the D3 dispersion correction.[10] The Karlsruhe all-electron triple-zeta basis set def2-TZVPP[11] was employed for all atoms. Geometry optimizations utilized the TIGHTOPT and TIGHTSCF criteria.

S6.2. Comparison of Metrical Parameters between Experimental and Calculated Structures.

Table S1. Comparison of metrical parameters for **[Fe(O)L^{OCH2O}]$^{-}$**

**Parameter**	**Experimental (XRD)**	**Calculated**
Fe–N_{amine}	2.096(2) Å	2.137 Å
Fe–N_{amide} (mean)	2.013(5) Å	2.026 Å
Fe=O	1.6415(19) Å	1.620 Å

Table S2. Comparison of metrical parameters for **[Fe(O)H$_3$buea]$^{-}$**

**Parameter**	**Experimental (XRD)**	**Calculated**
Fe–N$_{amine}$	2.0636(8) Å	2.094 Å
Fe–N$_{amide}$ (mean)	1.989(15) Å	2.006 Å
Fe=O	1.6804(7) Å	1.657 Å

Table S3. Comparison of metrical parameters for **[Fe(O)TMG$_3$tren]$^{-}$**

**Parameter**	**Experimental (XRD)**	**Calculated**
Fe–N$_{amine}$	2.112(3) Å	2.135 Å
Fe–N$_{amide}$ (mean)	2.005(5) Å	2.019 Å
Fe=O	1.661(2) Å	1.626 Å

S6.3. Evaluation of O–H Bond Strength

The thermodynamic parameters associated with transfer of an H-atom equivalent to **[Fe(O)L^{OCH2O}]$^{-}$** to give the ferric hydroxide **[Fe(OH)L^{OCH2O}]$^{-}$** were computationally determined by analyzing the hypothetical transfer of H• from TEMPOH. This method has recently been used by others to determine the element–hydrogen bond dissociation free energies (BDFEs) in metal-based systems.[12-14] Fully relaxed coordinates were used to perform an analytical frequency calculation using an expanded integration grid (GRIDX4). All frequency calculations on fully relaxed structures were confirmed to be devoid of imaginary vibrational frequencies.

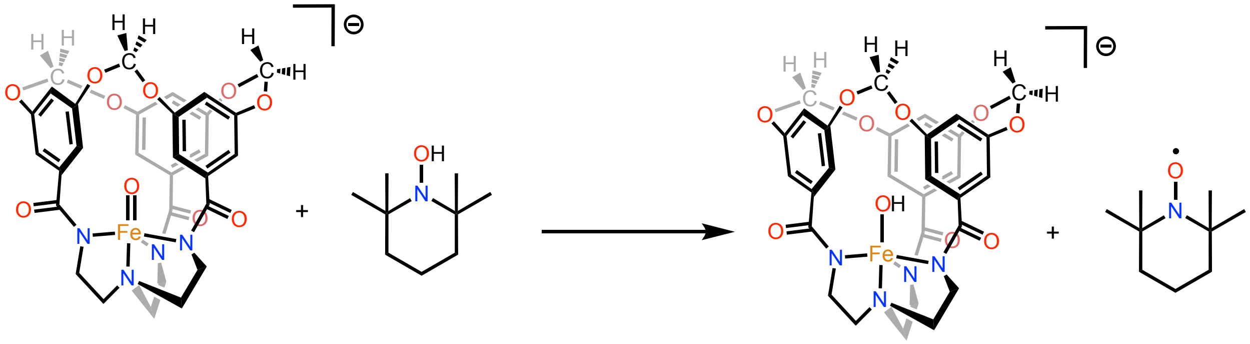

Scheme S1. H-atom transfer reaction used to computationally assess the strength of the O–H bond in [Fe(OH)L^{OCH2O}]$^{-}$.

Table S4. Energetic parameters obtained from single point and frequency calculations relevant to the above reaction. All energy values are listed in Hartrees (E_h). Gibbs free energy values (G) are determined at T = 298.15 K and P = 1.00 atm.

Molecule	Energy (gas)	*G (gas)*	Energy (MeCN)	$G_{correction}$ (MeCN)	*G (MeCN)*
**$[Fe(O)L^{OCH2O}]^-$**	–3394.2995	–3393.8102	–3394.3990	–0.0995	–3393.9097
**$[Fe(OH)L^{OCH2O}]^-$**	–3394.9449	–3394.4490	–3395.0422	–0.0973	–3394.5463
TEMPOH	–484.2560	–484.0183	–484.2626	–0.0066	–484.0249
TEMPO•	–483.6440	–483.4190	–483.6531	–0.0091	–483.4281

The Gibbs free energy change for reaction shown in Scheme S1 is therefore computed as –0.040 E_h (–24.8 kcal/mol) at 298.15 K. Given that the O–H BDFE of TEMPOH in acetonitrile has been experimentally determined to be 66 kcal/mol,[15] the Fe–O–*H* BDFE is thereby computed as 91 kcal/mol.

S6. Details of Crystallographic Structure Determinations

[K(Crypt)][1]. A crystal (0.4 x 0.269 x 0.181 mm^3) was placed onto a nylon loop and mounted on a Rigaku XtaLAB Synergy-S Dualflex diffractometer equipped with a HyPix-6000HE HPC area detector for data collection at 100.00(10) K. A preliminary set of cell constants and an orientation matrix were calculated from a small sampling of reflections.[16] A short pre-experiment was run, from which an optimal data collection strategy was determined. The full data collection was carried out using a PhotonJet (Mo) X-ray source with a frame time of 8.00 seconds and a detector distance of 34.0 mm. Series of frames were collected in 0.50º steps in ω at different 2θ, κ, and ϕ settings. After the intensity data were corrected for absorption, the final cell constants were calculated from the xyz centroids of 46334 strong reflections from the actual data collection after integration.[16] See Table S5 for additional crystal and refinement information.

The structure was solved using SHELXT[17] and refined using SHELXL.[18] The space group $Pca2_1$ was determined based on systematic absences and intensity statistics. Most or all non-hydrogen atoms were assigned from the solution. Refinement proceeded in an iterative fashion, with each stage including full-matrix least squares cycles, followed by a difference Fourier synthesis, which located any remaining electron density. All non-hydrogen atoms were refined with anisotropic displacement parameters. All hydrogen atoms were placed in ideal positions and refined as riding atoms with relative isotropic displacement parameters. The final full matrix least squares refinement converged to $R1 = 0.0381$ (F^2, $I > 2\sigma(I)$) and $wR2 = 0.0957$ (F^2, all data). The asymmetric unit contains one monoanionic Fe complex and one $[K(cryptand\text{-}222)]^+$ cation in general positions. The cation is modeled as disordered over two positions (0.58:0.42). The Fe atom is out the N2–N3–N4 plane, away from atom N1, by 0.2288(9) Å. Structure manipulation and figure generation were performed using Olex2.[19]

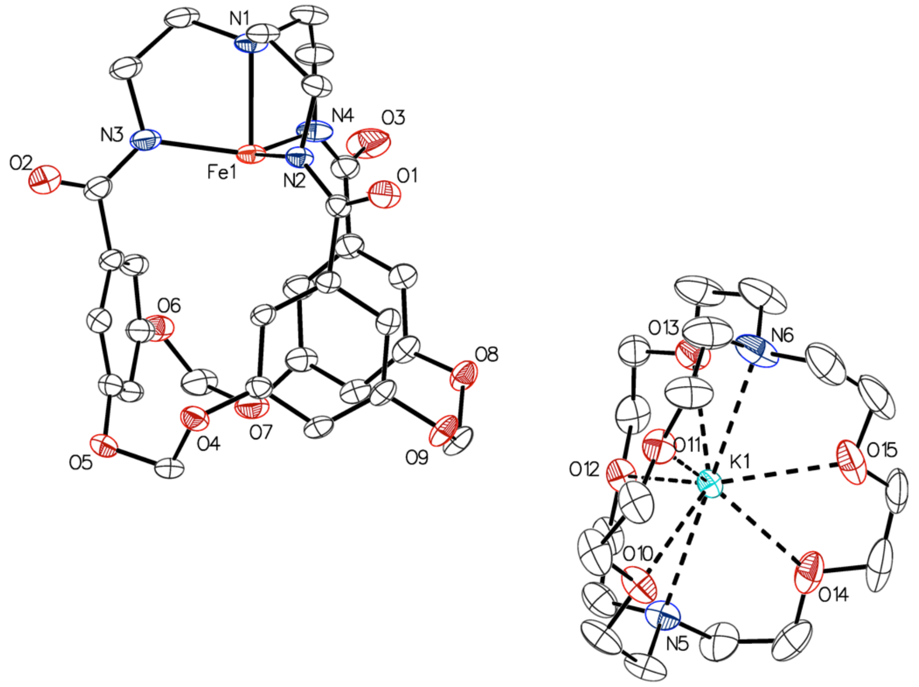

Figure S29. Solid-state structure of **[K(Crypt)][1]**. Hydrogen atoms and disorder in the cationic unit have been omitted for clarity. Selected bond distances (Å): Fe(1)–N(1) = 2.1471(15). Fe(1)–N(2) = 2.0375(15). Fe(1)–N(3) = 2.032(2). Fe(1)–N(4) = 2.030(2). Anisotropic displacement ellipsoids are drawn at the 50 % probability level.

[K(Crypt)][2]. A crystal (0.256 x 0.224 x 0.188 mm^3) was placed onto a nylon loop and mounted on a Rigaku XtaLAB Synergy-S Dualflex diffractometer equipped with a HyPix-6000HE HPC area detector for data collection at 100.00(10) K. A preliminary set of cell constants and an orientation matrix were calculated from a small sampling of reflections.[16] A short pre-experiment was run, from which an optimal data collection strategy was determined. The full data collection was carried out using a PhotonJet (Cu) X-ray source with frame times of 0.43 and 1.74 seconds and a detector distance of 34.0 mm. Series of frames were collected in 0.50º steps in ω at different 2θ, κ, and ϕ settings. After the intensity data were corrected for absorption, the final cell constants were calculated from the xyz centroids of 90513 strong reflections from the actual data collection after integration.[16] See Table S6 for additional crystal and refinement information.

The structure was solved using SHELXT[17] and refined using SHELXL.[18] The space group $Pca2_1$ was determined based on systematic absences and intensity statistics. Most or all non-hydrogen atoms were assigned from the solution. Full-matrix least squares / difference Fourier cycles were performed which located any remaining non-hydrogen atoms. All non-hydrogen atoms were refined with anisotropic displacement parameters. All hydrogen atoms were placed in ideal positions and refined as riding atoms with relative isotropic displacement parameters. The final full matrix least squares refinement converged to $R1 = 0.0345$ (F^2, $I > 2\sigma(I)$) and $wR2 = 0.0979$ (F^2, all data). The asymmetric unit contains one monocationic potassium complex and one monoanionic iron complex in general positions. The cation is

modeled as disordered over two positions (0.61:0.39). Structure manipulation and figure generation were performed using Olex2.[19]

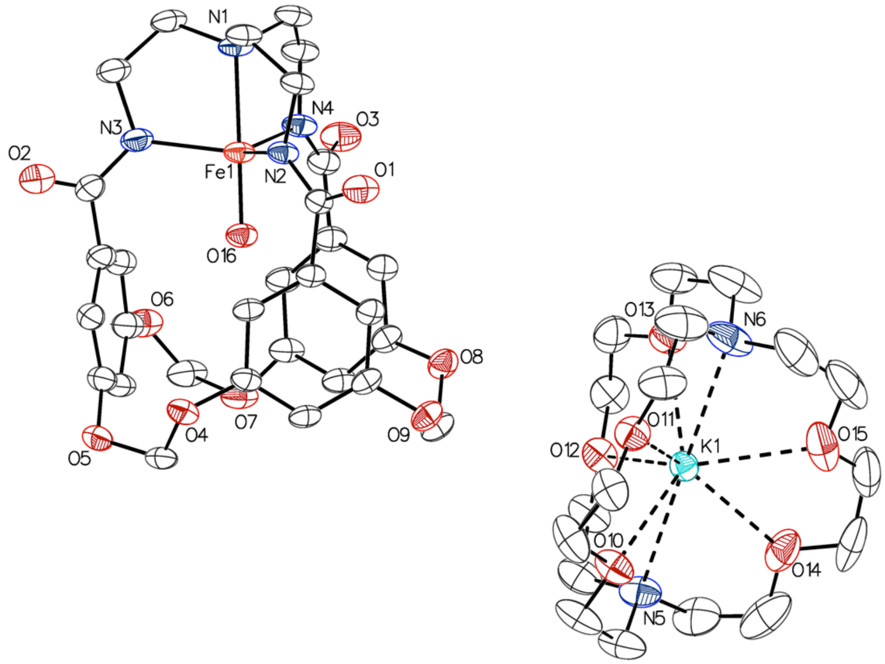

Figure S30. Solid-state structure of **[K(Crypt)][2]**. Hydrogen atoms and disorder in the cationic unit have been omitted for clarity. Selected bond distances (Å): Fe(1)–O(16) = 1.6415(19); Fe(1)–N(1) = 2.096(2); Fe(1)–N(2) = 2.018(2); Fe(1)–N(3) = 2.015(3); Fe(1)–N(4) = 2.005(3). Anisotropic displacement ellipsoids are drawn at the 50 % probability level.

Table S5. Crystal data and structure refinement for [K(Crypt)][**1**].

Identification code	brbcdh67	
Empirical formula	C48 H63 Fe K N6 O15	
Formula weight	1058.99	
Temperature	100.00(10) K	
Wavelength	0.71073 Å	
Crystal system	orthorhombic	
Space group	$Pca2_1$	
Unit cell dimensions	*a* = 15.9415(2) Å	α = 90°
	b = 11.8129(2) Å	β = 90°
	c = 26.6605(4) Å	γ = 90°
Volume	5020.58(13) $Å^3$	
Z	4	
Density (calculated)	1.401 Mg/m^3	
Absorption coefficient	0.457 mm^{-1}	
F(000)	2232	
Crystal color, morphology	colourless, block	
Crystal size	0.4 x 0.269 x 0.181 mm^3	
Theta range for data collection	1.528 to 33.213°	
Index ranges	$-22 \le h \le 22$, $-17 \le k \le 17$, $-40 \le l \le 37$	
Reflections collected	88271	
Independent reflections	16785 [*R*(int) = 0.0344]	
Observed reflections	14522	
Completeness to theta = 29.575°	100.0%	
Absorption correction	Multi-scan	
Max. and min. transmission	1.00000 and 0.88872	
Refinement method	Full-matrix least-squares on F^2	
Data / restraints / parameters	16785 / 364 / 884	
Goodness-of-fit on F^2	1.045	
Final *R* indices [*I*>2sigma(*I*)]	*R*1 = 0.0381, *wR*2 = 0.0923	
R indices (all data)	*R*1 = 0.0466, *wR*2 = 0.0957	
Absolute structure parameter	-0.008(3)	
Largest diff. peak and hole	0.482 and -0.250 e.$Å^{-3}$	

Table S6. Crystal data and structure refinement for [K(Crypt)][**2**].

Identification code	brbcdh61	
Empirical formula	C48 H63 Fe K N6 O16	
Formula weight	1074.99	
Temperature	100.00(10) K	
Wavelength	1.54184 Å	
Crystal system	orthorhombic	
Space group	$Pca2_1$	
Unit cell dimensions	a = 15.85548(5) Å	α = 90°
	b = 12.00738(5) Å	β = 90°
	c = 26.51712(11) Å	γ = 90°
Volume	5048.40(3) $Å^3$	
Z	4	
Density (calculated)	1.414 Mg/m^3	
Absorption coefficient	3.776 mm^{-1}	
F(000)	2264	
Crystal color, morphology	orange, block	
Crystal size	0.256 x 0.224 x 0.188 mm^3	
Theta range for data collection	3.333 to 80.387°	
Index ranges	$-20 \leq h \leq 20$, $-15 \leq k \leq 15$, $-33 \leq l \leq 33$	
Reflections collected	165248	
Independent reflections	10986 [R(int) = 0.0400]	
Observed reflections	10656	
Completeness to theta = 74.504°	100.0%	
Absorption correction	Multi-scan	
Max. and min. transmission	1.00000 and 0.81140	
Refinement method	Full-matrix least-squares on F^2	
Data / restraints / parameters	10986 / 304 / 893	
Goodness-of-fit on F^2	1.047	
Final R indices [I>2sigma(I)]	$R1$ = 0.0345, $wR2$ = 0.0970	
R indices (all data)	$R1$ = 0.0356, $wR2$ = 0.0979	
Absolute structure parameter	-0.0011(13)	
Largest diff. peak and hole	0.279 and -0.501 e.$Å^{-3}$	

S7. References

1. Armarego, L. L. F.; Chai, C. L. L. *Purification of Laboratory Chemicals, 5th ed.;* Elsevier, 2003.

2. Hastings, C. D.; Huffman, L. S. X.; Tiwari, C. K.; Betancourth, J. G.; Brennessel, W. W.; Barnett, B. R. Coordinatively Unsaturated Metallates of Cobalt(II), Nickel(II), and Zinc(II) Guarded by a Rigid and Narrow Void. *Inorg. Chem.* **2023**, *62*, 11920–11931.

3. Schardt, B. C.; Hill, C. L. Preparation of Iodobenzene Dimethoxide. A New Synthesis of [^{18}O]Iodosylbenzene and a Reexamination of Its Infrared Spectrum. *Inorg. Chem.* **1983,** *22*, *10*, 1563-1565.

4. Bismuto, A.; Müller, P.; Finkelstein, P.; Trapp, N.; Jeschke, G.; Morandi, B. One to Find Them All: A General Route to Ni(I)−Phenolate Species. *J. Am. Chem. Soc.* **2021**, *143*, 10642-10648.

5. Saltzman, H.; Sharefkin, J. G.; *Org.Synth.* **1963**, *43*, 60.

6. Huffman, L. S. X.; Seshadri, A.; Hastings, C. D.; Brennssel, W. W.; Franco, I.; Barnett, B. R. *ChemRxiv.* **2025**. DOI: 10.26434/chemrxiv-2025-cs0r1.

7. Neese, F. *Wiley Interdiscip. Rev.: Comput. Mol. Sci.* **2012**, *2,* 73.

8. Becke, A.D. A New Mixing of Hartree-Fock and Local Density-Functional Theories. *J. Chem. Phys.* **1993**, *98*, 1372–1377.

9. Lee, C.; Yang, W.; Parr, R. G. Development of the Colic-Salvetti Correlation-Energy into a Functional of the Electron Density Formula. *Phys. Rev. B: Condens. Matter Mater. Phys.* **1988**, *37*, 785–789.

10. Gimme, S.; Ehrlich, S.; Goerigk, L. Effect of the Damping Function in Dispersion Corrected Density Functional Theory. *J. Comp. Chem.* **2011**, *32*, 1456-1465.

11. Rappoport, D.; Furche, F. Property-optimized Gaussian Basis Sets for Molecular Response Calculations. *J. Chem. Phys.* **2010**, *133*, 134105.

12. Chen, S.; Rousseau, R.; Raugei, S.; Dupuis, M.; DuBois, D. L.; Bullock, R. M. Comprehensive Thermodynamics of Nickel Hydride Bis(Diphosphine) Complexes: A Predictive Model through Computations. *Organometallics.* **2011**, *30*, 6108–6118.

13. Bruch, Q. J.; Connor, G. P.; Chen, C.-H.; Holland, P. L.; Mayer, J. M.; Hasanayn, F.; Miller, A. J. M. Dinitrogen Reduction to Ammonium at Rhenium Utilizing Light and Proton-Coupled Electron Transfer. *J. Am. Chem. Soc.* **2019**, *141*, 20198–20208.

14. Niklas, J. E.; Otte, K. S.; Studvick, C. M.; Chowdhury, S. R.; Vlaisavljevich, B.; Bacsa, J.; Kleemiss, F.; Popov, I. A.; La Pierre, H. S. A Tetrahedral Neptunium(V) Complex. *Nat. Chem.* **2024**, *16*, 1490–1495.

15. Agarwal, R. G.; Coste, S. C.; Groff, B. D.; Heuer, A. M.; Noh, H.; Parada, G. A.; Wise, C. F.; Nichols, E. M.; Warren, J. J.; Mayer, J. M. Free Energies of Proton-Coupled Electron Transfer Reagents and Their Applications. *Chem. Rev.* **2022**, *122*, 1–49.

16. *CrysAlisPro*, version 171.43.136a; Rigaku Corporation: Oxford, UK, 2024.

17. Sheldrick, G. M. *SHELXT*, version 2018/2; *Acta. Crystallogr.* **2015**, *A71*, 3–8.

18. Sheldrick, G. M. *SHELXL*, version 2019/3; *Acta. Crystallogr.* **2015**, *C71*, 3–8.

19. Dolomanov, O. V.; Bourhis, L. J.; Gildea, R. J.; Howard, J. A. K.; Puschmann, H. *Olex2*, version 1.5; *J. Appl. Cryst.* **2009**, *42*, 339–341.
